# Genome‐wide association study reveals significant loci and candidate genes for fruit branch length in upland cotton

**DOI:** 10.1002/tpg2.70041

**Published:** 2025-05-29

**Authors:** Hui Chang, Honghu Ji, Ruijie Liu, Juling Feng, Jiayi Wang, Shuqi Zhao, Wei Li, Zehua Qiu, Nabil Ibrahim Elsheery, Shuxun Yu, Libei Li, Zhen Feng

**Affiliations:** ^1^ College of Advanced Agriculture Sciences Zhejiang A & F University Hangzhou China; ^2^ Jinhua Academy of Agricultural Sciences Jinhua Key Laboratory of Innovative Utilization of Special Grain Crops Resources in Central Zhejiang Province Jinhua China; ^3^ College of Agronomy Northwest A & F University Yangling China; ^4^ Huanggang Academy of Agricultural Sciences Huanggang China; ^5^ Agricultural Botany Department, Faculty of Agriculture Tanta University Tanta Egypt

## Abstract

The length of fruit branches significantly influences plant architecture in upland cotton (*Gossypium hirsutum* L.), which is crucial for optimizing fiber yield and quality. In this study, a comprehensive genome‐wide association study was conducted based on whole‐genome resequencing data that identified 249 significant SNPs associated with fruit branch length (FBL), forming 79 distinct quantitative trait loci (QTL) regions. Notably, stable QTL regions *qFBL‐A10‐4* and *qFBL‐D03‐17* were identified, harboring key candidate genes such as *Ghir_A10G014390* and *Ghir_D03G011390*. Superior haplotypes of these genes significantly enhance FBL, fiber yield, and quality, offering valuable targets for cotton breeding programs focused on optimizing plant architecture and productivity.

## Core Ideas

- A comprehensive genome‐wide association study identified 249 significant SNPs and 79 quantitative trait loci (QTL) regions associated with fruit branch length (FBL).
- Two stable QTL regions were discovered, containing candidate genes Ghir_A10G014390 and Ghir_D03G011390.
- Superior haplotypes of the identified genes were found to significantly increase FBL and improve cotton fiber trait.

**Abbreviations**
BLUP: best linear unbiased predictions
BRs: brassinosteroids
DREB: dehydration responsive element binding protein
FBA: fruit branch angle
FBL: fruit branch length
GWAS: genome‐wide association study
HNFFB: height of the node of the first fruit branch
IAA: indole‐3‐acetic acid
LD: linkage disequilibrium
LMM: linear mixed model
LRR‐RLKs: leucine‐rich repeat receptor‐like protein kinases
LRR‐RLP: leucine‐rich repeat receptor‐like protein
NFFB: node of the first fruit branch
PV: phenotypic variation
PVE: proportion of variance explained
qRT‐PCR: quantitative real‐time PCR
QTL: quantitative trait loci
SL: strigolactone
SLAF‐seq: specific‐locus amplified fragment sequencing
SSR: simple sequence repeat

## 1 INTRODUCTION

Upland cotton (*Gossypium hirsutum* L.) is a crucial source of natural textile fibers worldwide and holds significant importance in China's national economy (Z. J. Chen et al., 2007). The complexity of cotton cultivation and high labor costs have led to a decline in farmers’ enthusiasm for cotton planting, resulting in a gradual reduction in the planting area in recent years. The architecture of cotton plants plays a vital role in improving yield and quality (C. Q. Li, Song, Zhao, Wang, et al., 2014). By shaping an optimal plant architecture, it is possible to enhance the leaf area index, population light utilization efficiency, and per‐unit‐area yield, which are essential for achieving high yield and quality in cotton fibers (Wang & Li, 2008; Yu & Wang, 2012). Fruit branch length (FBL) (C. X. Wang et al., 2022) is a critical component in constructing an ideal plant architecture and is closely associated with the number of bolls per plant. The study of the analysis of genetic variability and path coefficient for yield contributing traits in advanced lines of cotton certificated that 10th sympodial branch length was positively and highly significantly correlated with total number of bolls per plant and ultimately correlated with seed cotton yield at genetic level (X. Y. Chen et al., 2010; Fatima et al., 2020). Boll number per plant was positively correlated with yield (Abro, 2010). Thus, a deep understanding of the genetic underpinnings of FBL and the identification of superior alleles related to FBL are imperative for the genetic improvement of cotton.

The architectural traits of upland cotton encompass a variety of characteristics such as plant height (PH), FBL, fruit branch angle (FBA), node of the first fruit branch (NFFB), height of the node of the first fruit branch (HNFFB), and others. These traits are complex quantitative traits controlled by multiple genes and are influenced by both genotype and environment. Previous studies have utilized simple sequence repeat (SSR) markers and bi‐parental mapping populations to perform quantitative trait loci (QTL) mapping for cotton architecture‐related traits. For instance, Li et al. used F_2_ and F_2:3_ populations derived from Baimian1 and TM‐1 to identify multiple QTLs associated with multiple plant architecture traits in cotton using SSR markers, and a total of 55 main‐effect QTLs (M‐QTLs) were detected. Four common M‐QTLs, qTFB‐10(F2/F2:3) for total fruit branches, qFBA‐5(F2/F2:3) for FBA, and qFBN‐26b(F2)/qFBN‐26(F2:3) for fruit branch nodes, were found, and seven major QTLs related to FBL, explaining 7.00%–17.00% of the phenotypic variation (PV) (C. Q. Li, Song, Zhao, Wang, et al., 2014). Similarly, B. H. Wang et al. (2006) detected three QTLs related to FBL (*qFBL‐20‐1*, *qFBL‐25‐1*, *qFBL‐25‐2*) using a recombinant inbred line population, explaining 9.06%, 7.56%, and 9.50% of the PV, respectively. In recent years, with the advancements in genomics, association analysis has been widely used in studies related to crop architecture. Map‐based cloning and genome‐wide association study (GWAS) have identified several genes controlling plant architectural traits. Su et al. were the first to utilize high‐density genetic markers for GWAS of cotton architectural traits, combining specific‐locus amplified fragment sequencing (SLAF‐seq) of 355 upland cotton accessions with phenotypic data from six environments with 30 significant SNPs identified, and four peak SNP loci located on chromosome D03 were simultaneously associated with PH, FBL, HNFFB, and NFFB. Furthermore, 21 candidate genes for plant architecture were predicted in a 0.95‐Mb region, including the four peak SNPs. One of these genes (*Gh_D03G0922*) was near the significant SNP rsD03_31584163 (8.40 kb), and its *Arabidopsis* homologs contain MADS‐box domains that might be involved in plant growth and development (Su et al., 2018). To reduce false positives caused by population structure in GWAS, Wang et al. employed 315 upland cotton accessions and 9244 high‐quality SNPs to conduct a restricted two‐stage multilocus genome‐wide association study using SLAF‐seq; they identified 157 SNPs significantly associated with plant architecture, with 55 SNPs significantly associated with FBL, mainly distributed on chromosomes A02, A04, A05, A06, A08, A09, D01, D02, D03, D06, and D12; additionally, they discovered four major loci (LDB_1_21982826, LDB_9_51578589_51578819, LDB_16_37952328, and LDB_19_52309050_52309284) co‐located for PH and FBL (C. X. Wang et al., 2022). Although previous association analyses using different mapping populations have identified several QTL regions and genetic loci significantly associated with FBL, studies utilizing resequencing data to elucidate the genetic characteristics of FBL have not yet been reported. This study aims to fill this gap by performing a comprehensive GWAS on FBL in 355 upland cotton accessions based on resequencing data.

Currently, several genes involved in the regulation of branch development have been reported across various crops. The development of branches is influenced by hormones such as gibberellins, indole‐3‐acetic acid (IAA), brassinosteroids (BRs), and strigolactones (SLs) (B. Wang et al., 2018). In rice, *PANICLE RACHIS LENGTH5* (*Prl5*) encodes a gibberellin biosynthesis enzyme, which is associated with the elongation of the panicle rachis (Agata et al., 2020). In cotton, reduced expression of GA synthesis genes inhibits cell elongation, leading to decreased PH and shorter internodes of fruit branches (L. Wang et al., 2014). Additionally, IAA mediates the expression of *BRC1* by controlling the antagonistic factors cytokinin  and SL (Drummond et al., 2015). *AtBRC1* and *AtBRC2* were involved in branch regulation, encoding TCP (for Teosinte branched1, Cycloidia, and Proliferating cell factor) transcription factors that negatively regulate branch growth and development. *AtBRC1* plays a more crucial role in branch development compared to *AtBRC2* (Aguilar‐Martínez et al., 2007). Similarly, SL inhibits branch development by regulating the expression of IAA‐related genes (Crawford et al., 2010). Moreover, branch development is also regulated by other genes. For example, the dehydration responsive element binding protein (DREB), part of the AP2/EREBP transcription factor family, has been shown to influence branch traits in cotton. Overexpression of *GhDREB1B* leads to significant reductions in PH and FBL, as well as a decrease in FBA (Ji et al., 2021). Liu et al. found that *GhCEN* is highly expressed in the axillary buds and shoot apices of cotton. Overexpression of this gene delays the transition from vegetative to reproductive growth, while RNA interference silencing of *GhCEN* accelerates flowering time and shortens fruit branches (Liu et al., 2018). Y. C. Zhang et al. (2018) identified the *Arabidopsis thaliana Centroradialis* gene in an F_2_ population derived from a cross between short‐branching X1570 and long‐branching Ekangmian13, which is involved in the regulation of fruit branch development.

Although previous studies have investigated the architectural traits of cotton, a comprehensive GWAS on the genetic basis of FBL in cotton remains lacking. This study aims to fill this gap by performing a GWAS using resequencing data from 355 upland cotton accessions. Our objective is to identify genetic loci associated with FBL and to pinpoint superior alleles that regulate this trait. The findings from this research will provide a robust foundation for future gene cloning efforts and genetic improvement of FBL, thereby offering critical theoretical support for breeding programs aimed at optimizing cotton plant architecture.

**Core Ideas**

- A comprehensive genome‐wide association study identified 249 significant SNPs and 79 quantitative trait loci (QTL) regions associated with fruit branch length (FBL).
- Two stable QTL regions were discovered, containing candidate genes Ghir_A10G014390 and Ghir_D03G011390.
- Superior haplotypes of the identified genes were found to significantly increase FBL and improve cotton fiber trait.

## 2 MATERIALS AND METHODS

### 2.1 Experimental materials and field planting

This study involved 355 accessions of upland cotton germplasm, representing a broad spectrum of breeding achievements from the pre‐1950s to the 2020s. Specifically, 10 accessions originated from the initial stages of cotton breeding before the 1950s. Thirty‐four accessions were from the 1950s to the 1970s, a period characterized by efforts to increase fiber yield. Seventy‐two accessions came from the 1980s to the 1990s, targeting improvements in fiber yield, quality, and disease resistance. Finally, 187 accessions from the 2000s to the 2020s were selected for advancements in fiber quality, the incorporation of transgenic technologies, and suitability for mechanized harvesting. These germplasm accessions were planted in two consecutive years in Liaocheng, Shandong Province (36°48′ N, 115°41′ E), Huanggang, Hubei Province (30°57′ N, 114°92′ E), and Sanya, Hainan Province (18°36′ S, 109°17′ E). The study was conducted across six different environmental settings: E1 (Liaocheng‐2021), E2 (Huanggang‐2021), E3 (Sanya‐2021‐2022), E4 (Liaocheng‐2022), E5 (Huanggang‐2022), and E6 (Sanya‐2022‐2023). All field management practices were adapted to local agricultural standards.

### 2.2 Phenotypic characterization and statistical analysis

At the boll‐opening phase, we used a ruler with high precision to make direct measurements from the base of the fruit branch (where it connects with the main stem) to the tip of the fruit branch to record the length. In order to obtain more accurate and representative results, we chose to measure the fourth, fifth, and sixth intermediate fruit branches from the top of the plant down and use the average of these measurements to represent the phenotypic value of each plant. We also ensured that the ruler is parallel to the fruit branch to avoid tilting the measurement results. In six environments, we set up at least two repetitions. To ensure data reliability and measurement consistency, six plants with similar growth conditions were measured and averaged for each replicate. To mitigate the influence of environmental variables on the phenotypic data, the best linear unbiased predictions (BLUP) across six different environmental conditions were calculated using the R package “lme4” (Bates et al., 2015). Additionally, descriptive statistical analyses were conducted using the “pastecs” package (https://github.com/SciViews/pastecs), which included the computation of maximum, minimum, and mean values; coefficient of variation; skewness; and kurtosis. The distribution of phenotypic frequencies was visualized using the “ggplot2” package (Wickham, 2011). Correlations between environments were analyzed using the “corrplot” package (Wei et al., 2017), and the selection of elite varieties was achieved through cluster analysis using the “ggtree” package (G. C. Yu et al., 2017).

### 2.3 GWAS and visualization

In this investigation, we utilized a set of 355 accessions that underwent whole‐genome resequencing (PRJNA389777) (https://www.ncbi.nlm.nih.gov/sra) to develop SNP markers, facilitate variant detection, and analyze population structure (L. B. Li et al., 2023, 2020, 2024). SNP filtration was performed using VCFtools (Danecek et al., 2011) (version 0.1.1.6), retaining 2,262,367 high‐quality SNPs with a minor allele frequency >0.05 and a missing rate <0.2. TM‐1 (v HAU_v1.1) (M. J. Wang et al., 2019) served as the reference genome for further analyses of these SNPs. GWAS was conducted using a linear mixed model (LMM) approach via GEMMA (Vogt et al., 2022) (version 0.98.5), assessing both the phenotypic values from six different environments and their BLUP. The significance threshold was initially determined by Bonferroni correction (Noble, 2009), calculated as *p* = 1/*n* (where *n* is the number of SNPs), yielding a stringent threshold of *p* = 4.42E‐07. Given the potential for overlooking significant candidate loci under this threshold, it was adjusted to 1E‐05 (−Log10(*p*) = 5) to capture a wider array of significant SNPs. The proportion of variance explained (PVE) was calculated following methodologies described in previous studies (Feng et al., 2022). Visualization of the GWAS results, including Manhattan plots and quantile–quantile plots, was conducted using the R package “CMplot” (Yin et al., 2021).

### 2.4 Haplotype analysis and candidate gene prediction

In this study, QTL identified across multiple environments were considered stable intervals (X. Hu et al., 2024). Linkage disequilibrium (LD) analysis was performed on these stable intervals and surrounding regions using Haploview (Barrett et al., 2005; Su et al., 2020) (version 4.2) to identify critical LD blocks and candidate genes controlling FBL. The “LDheatmap” package (Shin et al., 2006) was employed to visualize LD patterns as heatmaps. Candidate genes and their corresponding protein sequences were aligned against the Arabidopsis annotation database (https://www.arabidopsis.org/) to obtain gene annotation information. Haplotype analysis of candidate genes was conducted using the “ggplot2” package (Wickham, 2011). Downloaded National Center for Biotechnology Information (NCBI) Sequence Read Archive (SRA) database (PRJNA257154) (https://www.ncbi.nlm.nih.gov/bioproject/) wild sequencing data, cultivated and wild type single times frequency change. RNA‐seq data from the NCBI SRA database (PRJNA490626) (https://www.ncbi.nlm.nih.gov/bioproject/) were utilized to assess the expression levels of candidate genes across different tissues. Additionally, genetic diversity across different breeding periods was calculated using VCFtools (Danecek et al., 2011).

### 2.5 RNA extraction and quantitative real‐time PCR analysis

To further validate the expression levels of candidate genes across different haplotypes, quantitative real‐time PCR (qRT‐PCR) analysis was conducted on two selected genes. Gene sequences for these candidate genes were retrieved from CottonGen (https://www.cottongen.org/), and gene‐specific primers were designed using the NCBI resource (https://www.ncbi.nlm.nih.gov/tools/primer‐blast/index.cgi?LINK_LOC=BlastHome). These primers are listed in Table S1. The plants exhibiting large FBL CPB12‐1‐7 and Xinluzhong34, along with those exhibiting small FBL, Han9609 and CRI30, were cultivated in Sanya, Hainan Province (18°36′ S, 109°17′ E). In January 2024, axillary buds from each cultivar were sampled and immediately frozen in liquid nitrogen, then stored at −80°C. Total RNA was extracted using the FastPure Universal Plant Total RNA Isolation Kit (RC411‐01, Vazyme). High‐quality RNA was utilized for cDNA synthesis with three biological replicates, performed using the HiScript II Q RT SuperMix for qPCR (R223) kit. The quantitative PCR was carried out using Vazyme's Taq Pro Universal SYBR qPCR Master Mix. Gene expression levels were quantified using the 2^−ΔΔCt^ method (Livak & Schmittgen, 2001), with three biological replicates performed for each sample.

## 3 RESULTS

### 3.1 Phenotypic characterization of FBL

**FIGURE 1:**
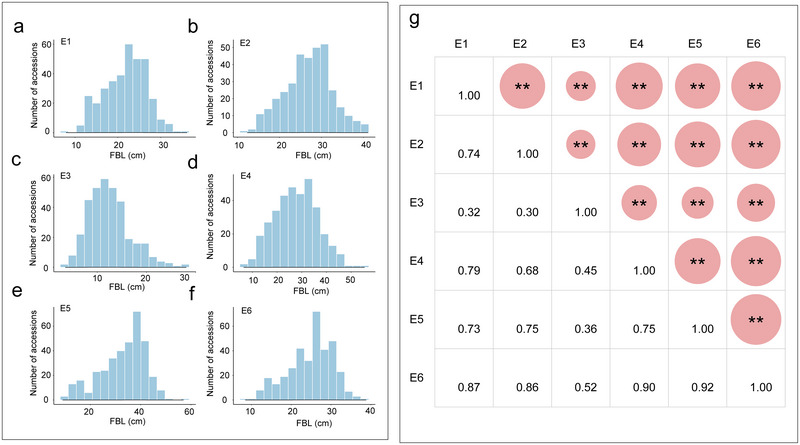
Phenotypic variation analysis of fruit branch length (FBL). (a–f) Distribution of FBL in six environments (E1: Liaocheng‐2021; E2: Huanggang‐2021; E3: Sanya‐2021‐2022; E4: Liaocheng‐2022; E5: Huanggang‐2022; and E6: Sanya‐2022‐2023). (g) Correlation analysis of FBL in six environments, with significance levels indicated (*p < 0.05, **p < 0.01). Each asterisk (*) in the upper right corresponds one‐to‐one with the value in the lower left.

The frequency distribution of FBL across six environmental settings is illustrated in histograms (Figure 1a–f). Correlation coefficients between environments ranged from 0.30 to 0.92, with the lowest correlation observed between E2 and E3, and the highest between E5 and E6 (Figure 1g). In the environment of E3, due to the large rainfall and short sunshine duration during the growth period of fruit branches, the development of fruit branches is hindered to a certain extent, resulting in differences between the environments. Descriptive statistical analyses of FBL across these environments revealed a range from 4.06 cm to 57.04 cm (Tables S2 and S3). The minimum value was recorded in E3, while the maximum occurred in E5. Coefficients of variation ranged from 21.22% to 34.90%, indicating substantial variability within and across environments (Table S3). To better understand the underlying genetic architecture of FBL, the phenotypic data were further subjected to a normality test, revealing that while some environments exhibited normal distribution, others showed slight skewness and kurtosis (Table S3). Broad‐sense heritability for FBL was estimated at 65.25%, suggesting a strong genetic control (Table S3). Analysis of variance (ANOVA) was conducted to explore the effects of genotype‐environment interactions on FBL, revealing significant influences (*p *< 0.001) (Table S4). The considerable variation in FBL, coupled with high heritability and significant genotype‐environment interactions, underscores the potential for further GWAS.

### 3.2 GWAS analysis of FBL

**FIGURE 2:**
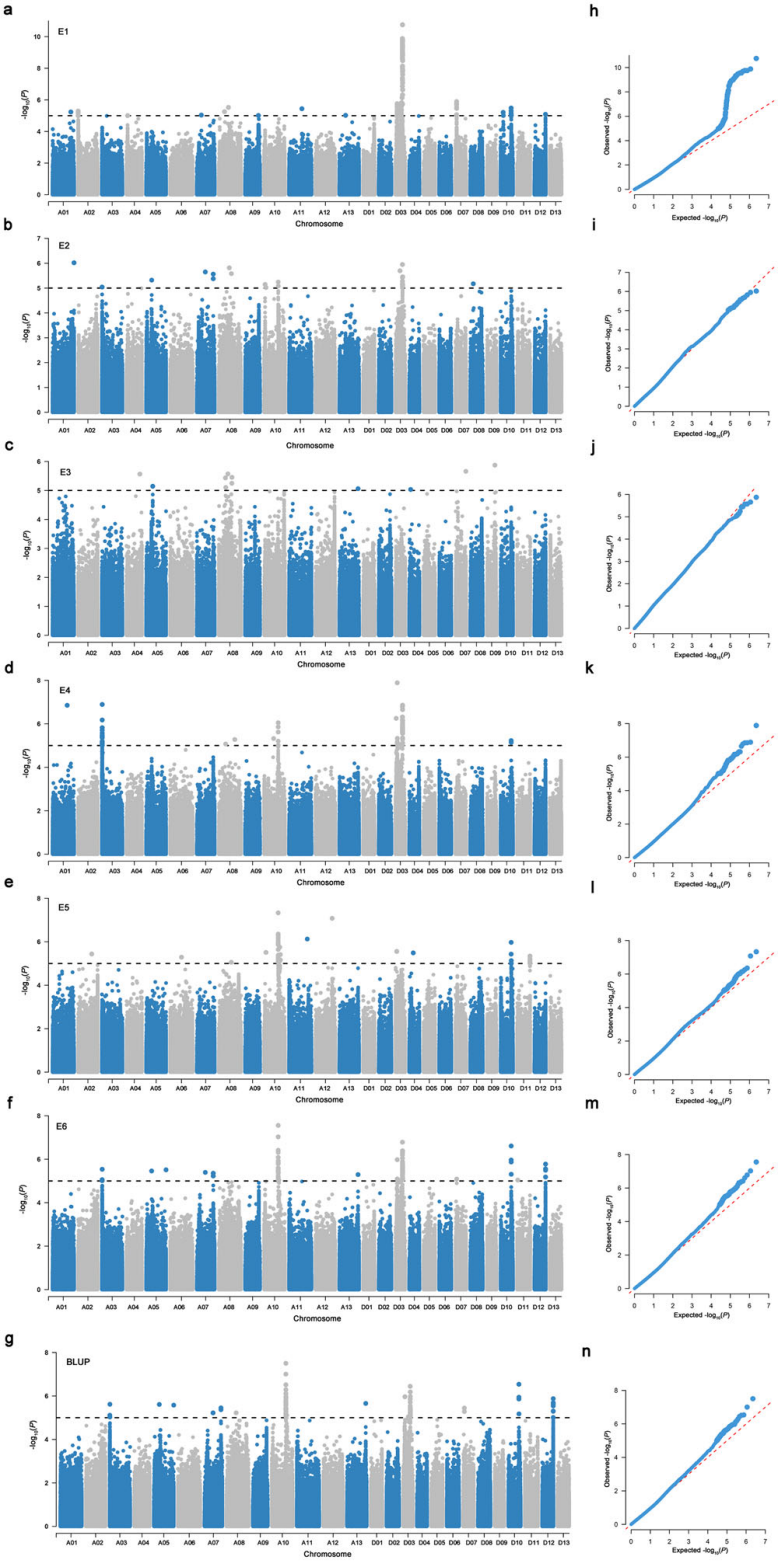
Genome‐wide association study result for fruit branch length (FBL) in E1–E6 and best linear unbiased predictions (BLUP). (a–g) Manhattan plots of FBL in E1–E6 and BLUP. The horizontal black dashed line represents the significance threshold of −Log10(*p*) = 5. (E1: Liaocheng‐2021; E2: Huanggang‐2021; E3: Sanya‐2021‐2022; E4: Liaocheng‐2022; E5: Huanggang‐2022; E6: Sanya‐2022‐2023). (h–n) quantile–quantile (*Q‐Q*) plots for FBL, respectively.

The LMM method was employed to conduct a GWAS on FBL. A significance threshold of −Log_10_(*p*) = 5 was set, identifying 249 significant SNPs across environments E1–E6 and BLUP values (Figure 2). The range of −Log10(*p*) values spanned from 5.00 to 10.76, and the PVE by these SNPs ranged from 5.33% to 11.96%. SNPs were classified into 79 distinct QTLs based on a 300 Kb interval (Table S5). Notably, 88 of these SNPs were identified in at least two environmental conditions, spanning 15 QTL regions, and are considered stable SNPs. These stable SNPs predominantly clustered in two regions: *qFBL‐A10‐4* and *qFBL‐D03‐17*. *The qFBL‐A10‐4* region contained 26 significant SNPs, all localized in at least two environments, with −Log10(*p*) values ranging from 5.09 to 7.56 and PVE values from 5.42% to 8.30%. The *qFBL‐D03‐17* region harbored 41 significant SNPs, with −Log10(*p*) values from 5.28 to 10.76 and PVE values from 5.69% to 11.96%, 31 of which were stable across multiple environments. Given these findings, *qFBL‐A10‐4* and *qFBL‐D03‐17* are designated as stable QTL regions. Subsequent analyses will focus on candidate gene identification within these regions.

### 3.3 Identification of candidate genes for FBL on chromosome A10

**FIGURE 3:**
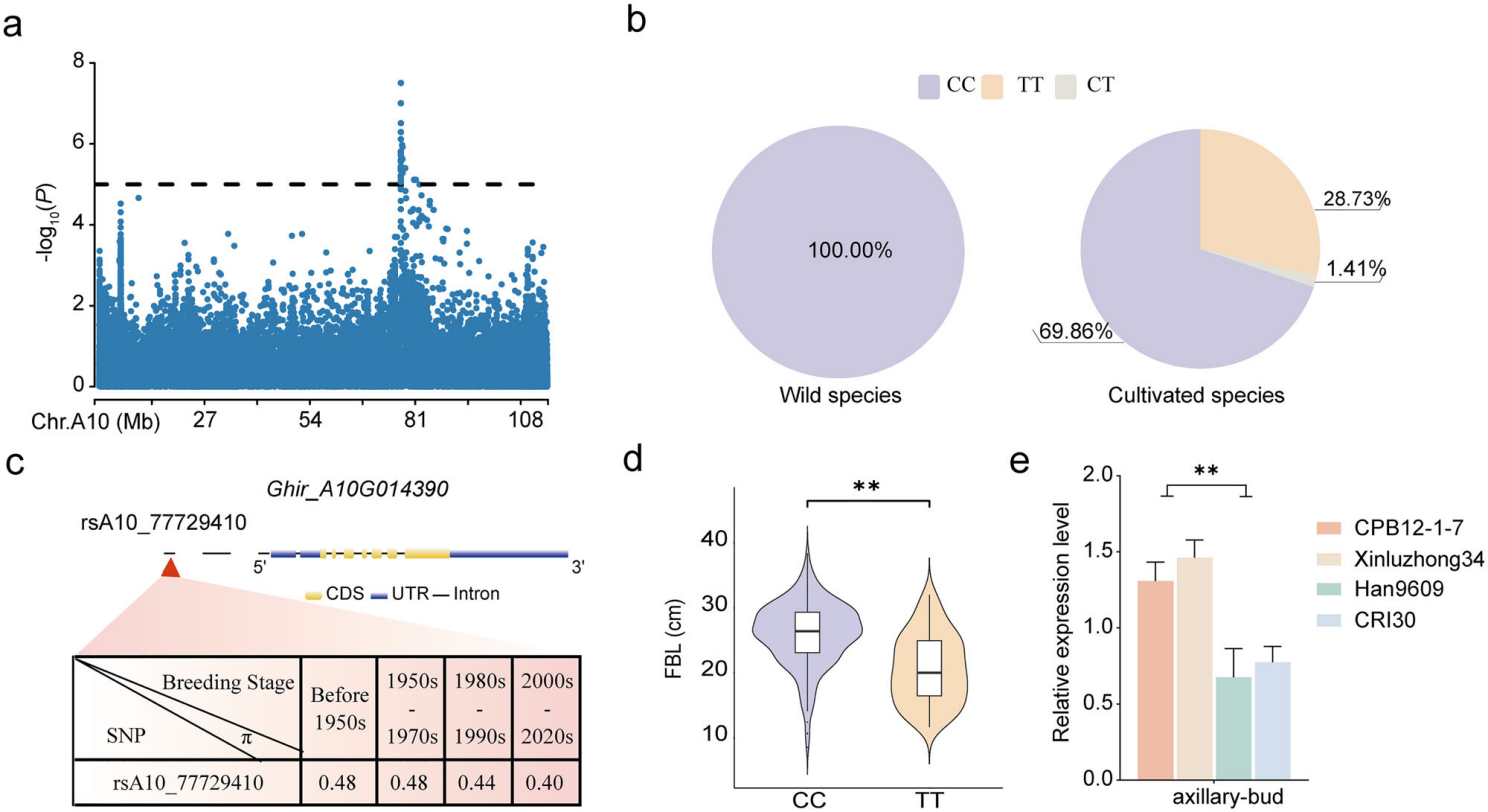
Identification of candidate genes in the *qFBL‐A10‐4* quantitative trait loci (QTL) interval on chromosome A10. (a) Local Manhattan plots displaying fruit branch length (FBL)‐related genes on chromosome A10 in best linear unbiased predictions (BLUP). (b) Differentiation in the genetic diversity distribution of significant SNP rsA10_77729410 between wild and cultivated species, with different haplotypes. (c) Gene structure and diversity across four breeding stages. (d) Violin plots for FBL across two haplotypes (**p < 0.01). (e) Expression level analysis of *Ghir_A10G014390* between CPB12‐1‐7 and Xinluzhong34, which carried haplotype CC, and Han9609 and CRI30 which carried haplotype TT (**p < 0.01).

The candidate region *qFBL‐A10‐4* contains a significant cluster of SNPs as depicted in Figure 3a. LD analysis confined the *qFBL‐A10‐4* to a linkage block spanning 500,391 bp according to the TM‐1 (v HAU_v1.1) reference genome (M. J. Wang et al., 2019). Within this interval, 10 genes are identified (Table S6), including *Ghir_A10G014370*, *Ghir_A10G014380*, and *Ghir_A10G014390*, all annotated as leucine‐rich repeat receptor‐like protein kinases (LRR‐RLKs). LRR‐RLKs play a crucial role in plant growth and development (Steidele & Stam, 2021), with several genes in this family implicated in regulating the development of meristematic tissues. For example, CLAVATA1, CLAVATA2, and CLAVATA3 are involved in the CLAVATA signaling pathway that maintains shoot apical meristem development (Chou et al., 2016). Transcriptomic data from TM‐1 across various tissues (Hu et al., 2019) revealed that while *Ghir_A10G014370* and *Ghir_A10G014380* show no expression in any tissue, *Ghir_A10G014390* is mainly expressed in stems, leaf, sepal, bract, ovule, and fiber development period. Homology comparison identified Ghir_A10G014390's ortholog in *Arabidopsis* as AtRLP15. The SNP rsA10_77729410 is located 2128 bp upstream of the gene and exhibits two haplotypes: CC and TT. No SNPs have been detected within the exonic or intronic regions of this gene. Comparisons of haplotype frequencies between semi‐wild and cultivated accessions revealed a decrease in the CC haplotype frequency in cultivated varieties compared to semi‐wild upland cotton (Figure 3b), and a reduction in genetic diversity in accessions post‐1980s compared to pre‐1980s (Figure 3c), suggesting artificial selection in this region. Haplotype analysis showed that accessions containing the CC haplotype significantly exhibited greater FBL than those with the TT haplotype (Figure 3d). Furthermore, based on published phenotype data on FBA, fiber quality, and boll weight (BW) (Feng et al., 2022; L. B. Li et al., 2024; Su et al., 2016), it was determined that while this gene does not significantly impact FBA (Figure S1a), accessions with the CC haplotype significantly outperformed TT‐containing accessions in terms of fiber length (FL), fiber elongation (FE), fiber strength (FS), fiber uniformity (FU), and BW (Figure S2). Quantitative RT‐PCR analysis revealed that the expression level of this gene in accessions containing the CC haplotype was significantly higher than in those with the TT haplotype (Figure 3e). Through the above empirical results, we inferred that *Ghir_A10G014390* on chromosome A10 has potential role responsible for improving fiber quality traits and may be beneficial to cotton breeding.

### 3.4 Identification of candidate genes for FBL on chromosome D03

**FIGURE 4:**
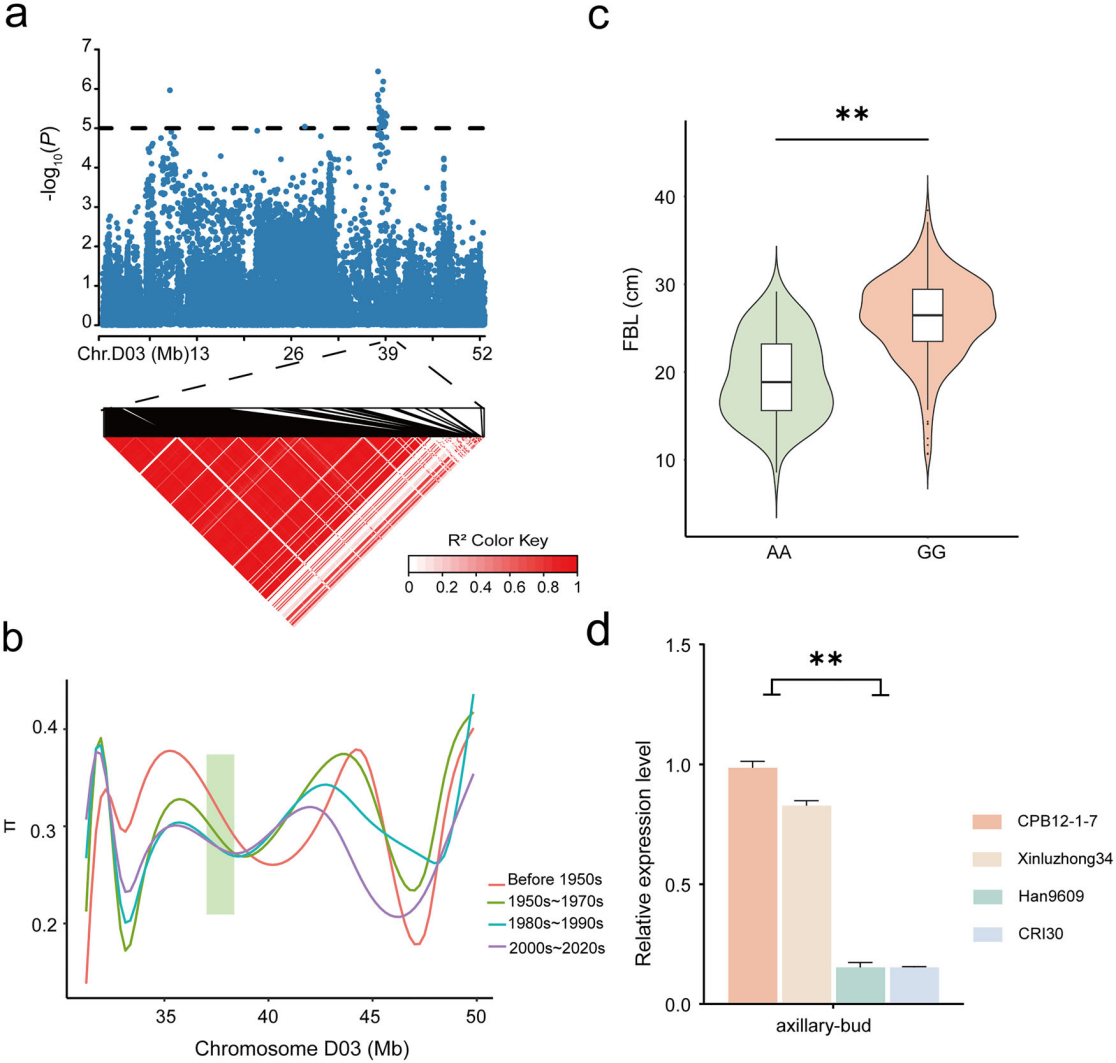
Identification of candidate genes in the *qFBL‐D03‐17* quantitative trait loci (QTL) interval on chromosome D03. (a) Local Manhattan plots displaying fruit branch length (FBL)‐related genes and a linkage disequilibrium heatmap within candidate region on chromosome D03. (b) Diversity of QTL region across four breeding stages, with strongly linked regions highlighted in green. (c) Violin plot for haplotype analysis of significant rsD03_39307341 (**p < 0.01). (d) Expression level analysis of *Ghir_D03G011390* between CPB12‐1‐7 and Xinluzhong34 which carried haplotype GG, and Han9609 and CRI30, which carried haplotype AA (**p < 0.01).

The candidate region *qFBL‐D03‐17*, consistently identified across multiple environments (E1, E2, E4, E6, and BLUP), spans a linkage interval of 1,667,289 bp (Figure 4a). A notable reduction in genetic diversity within this QTL has been observed over successive breeding periods (Figure 4b), highlighting selective pressures potentially exerted during cotton breeding. According to the TM‐1 (v HAU_v1.1) reference genome (M. J. Wang et al., 2019), 49 genes have been annotated within this interval (Table S7). Among these, *Ghir_D03G011390* is orthologous to *AtREF6* in *Arabidopsis*, a gene whose mutation is known to result in BR‐related phenotypes, including impaired cell elongation and reduced expression of BR target proteins (X. F. Yu et al., 2008). No SNPs were found within *Ghir_D03G011390*; however, a significant SNP, rsD03_39307341 located at 22,137 bp upstream of the gene *Ghir_D03G011390*, exhibits two haplotypes: AA and GG. Accessions with the GG haplotype show significantly greater FBL compared to those with the AA haplotype (Figure 4c). Furthermore, expression levels of this gene are significantly higher in GG haplotype accessions than in AA (Figure 4d), suggesting that regulatory elements affecting gene expression might be located near this SNP. Interestingly, this QTL was also identified in our previous studies on early maturity traits (L. B. Li et al., 2020), indicating its potential involvement in multiple phenotypic expressions. Further analysis of published data on FBA and fiber quality and yield‐related traits (Feng et al., 2022; L. B. Li et al., 2024; Su et al., 2016) reveals that accessions with the GG haplotype have significantly lower FBA compared to those with the AA haplotype (Figure S1b). However, they exhibit significantly greater FL, FE, FU, and BW (Figure S3).

### 3.5 Impact of superior haplotypes on cotton fiber quality and yield

**FIGURE 5:**
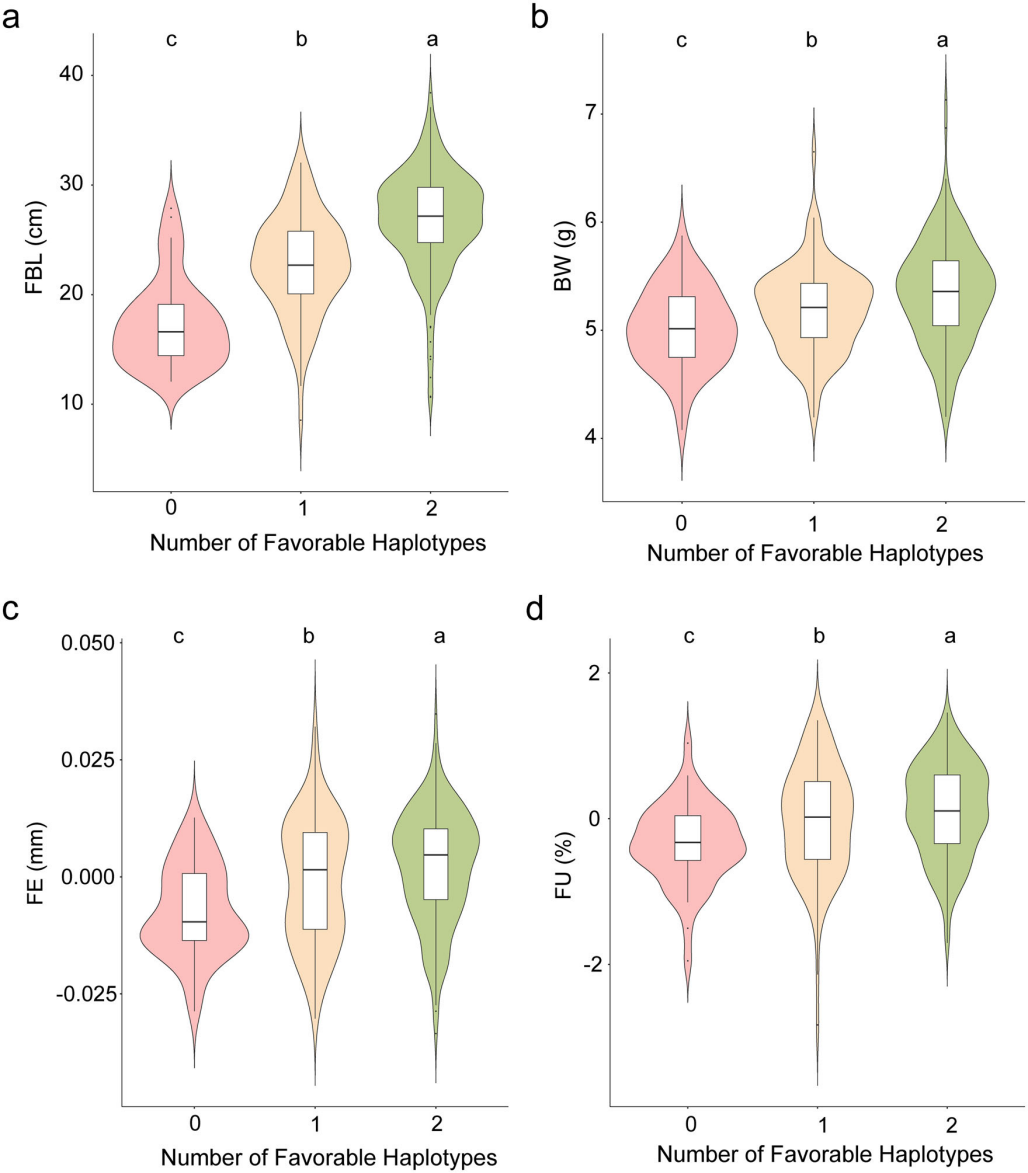
Effect of favorable allelic aggregation on phenotype. (a) Violin plot for fruit branch length (FBL) plotted as the favorable haplotype number. (b) Violin plot for boll weight (BW) plotted as the favorable haplotype number. (c) Violin plot for fiber elongation (FE) plotted as the favorable haplotype number. (d) Violin plot for fiber uniformity (FU) plotted as the favorable haplotype number.

Plant architecture is a critical trait influencing cotton fiber yield and quality. To explore the cumulative effects of two candidate genes on these traits, an ANOVA analysis was performed on phenotypes associated with superior haplotypes of these genes. Based on the presence of superior haplotypes, the 355 germplasm accessions were categorized into three distinct groups (Table S8): double superior haplotypes (CC‐GG), single superior haplotype (CC‐AA/TT‐GG), and no superior haplotypes (TT‐AA). The results revealed that accessions with both superior haplotypes exhibited significantly higher FBL, BW, FE, and FU compared to those with a single superior haplotype, and these traits were markedly superior in the double superior haplotype group compared to those without any superior haplotypes (Figure 5a–d). This suggests that the cumulative effect of these superior haplotypes significantly enhances FBL and overall cotton fiber yield and quality.

## 4 DISCUSSION

FBL is a pivotal trait in cotton breeding that significantly impacts the overall architecture, yield, and adaptability of the cotton plant (Donald, 1968; Kaggwa‐Asiimwe et al., 2013). The findings from this GWAS study, which identified several SNPs associated with FBL and highlighted stable QTL regions on chromosomes A10 and D03, underscore the complexity and importance of this trait in upland cotton. FBL directly influences the plant's architecture, affecting how efficiently it can capture sunlight and thus impacting photosynthesis and overall biomass production (Chapepa et al., 2020; L. B. Li et al., 2024). Longer fruit branches may favor more buds and the number of bolls, thus affecting the final yield. However, overlong fruit branches may also lead to plant structural instability, affecting photosynthetic efficiency and resource allocation, which in turn may negatively affect fiber quality. Optimal branch length is crucial for maximizing light interception within the plant canopy, which is essential for higher yield potential (Zhong et al., 2024). In addition to genetic factors, the optimal length of FBL may be affected by planting density, cultivation methods, and other aspects. In the future, control variable tests can be further designed to find the optimal length of FBL. Furthermore, the length of fruit branches determines the spatial arrangement of cotton bolls, influencing both the number and the size of the bolls, and ultimately, the yield per plant (X. Y. Chen et al., 2010; Fatima et al., 2020). In addition to its impact on yield, FBL is also closely linked to fiber quality traits. The genetic loci associated with branch length may have pleiotropic effects on fiber length, strength, and fineness. Breeding for optimal FBL could, therefore, contribute not only to enhanced yields but also to the production of high‐quality cotton fibers, which are critical for meeting market demands.

This study identifies 249 significant SNPs associated with FBL in cotton, distributed across the At and Dt subgenomes, with no significant subgenomic preference. Notably, chromosome D03 harbors the highest number of significant SNPs (77), corroborating previous studies (C. Q. Li, Song, Zhao, Wang, et al., 2014; C. Q. Li, Song, Zhao, Xia, et al., 2014; Mei et al., 2017; Su et al., 2018) that have also identified candidate regions on D03 related to plant architecture traits such as PH, FBL, and the NFFB. These findings suggest that D03 is a critical chromosomal region for regulating FBL, as well as other related traits in cotton. The number of significant SNPs on D03 is similar to earlier reports. For instance, prior GWAS analyses (L. B. Li et al., 2020) and QTL mapping efforts have pinpointed D03 as a key locus for traits affecting plant structure and yield, such as PH and early maturity traits (C. X. Wang et al., 2023). The high consistency of SNPs identified in this study with those reported in previous research highlights the robustness of D03 as a hotspot for traits crucial to cotton productivity. This suggests that D03 harbors a cluster of genes that play vital roles in the regulation of growth and development in cotton, making it a prime target for further genetic exploration and breeding programs.

So far, most studies on FBL have used traditional SSR molecular markers (C. Q. Li, Song, Zhao, Wang, et al., 2014; B. H. Wang et al., 2006); however, SSR markers have limitations in terms of marker density and resolution, which can hinder the discovery of finer genetic associations. While Su et al. (2018) used high‐density mapping for GWAS based on simplified sequencing, it represented an advancement in the field. In contrast to the previous studies, 2,262,367 high‐quality SNPs were used for whole gene association analysis of FBL based on resequencing technology in this study, and more abundant genetic loci were mined, significantly increasing the marker density compared to earlier studies. In addition, based on the data from previous studies (Feng et al., 2022; L. B. Li et al., 2024), this study also made a preliminary analysis of the effects of the candidate genes on cotton yield. These provide new clues for cotton plant type breeding and yield improvement. We compared our results with published studies based on SNP and SSR markers. Among the significant loci identified, SNPs rsA12_90699572 overlap with previously reported QTLs (C. Q. Li, Song, Zhao, Wang, et al., 2014). In addition, five SNPs, including rsD03_33921827, rsD03_37595985, rsD03_37966392, rsD03_38030754, and rsD03_38124641, were consistent with those reported by Su et al. (2018). Among these, rsD03_37595985, rsD03_37966392, rsD03_38030754, and rsD03_38124641 have been located in multiple environments. That suggests their potential role in controlling FBL and possibly influencing other agronomically important traits like fiber quality and yield.

In this study, *Ghir_A10G014390* and *Ghir_D03G011390* were identified as major candidate genes influencing FBL in cotton. *Ghir_A10G014390* was annotated on chromosome A10, belonging to the leucine‐rich repeat receptor‐like protein (LRR‐RLP) family, and identified as candidate genes associated with altered plant architecture and meristem development. In previous reports, this family has been shown to play significant roles in plant growth, development, and defense mechanisms. For example, CLAVATA2 (RLP10) is involved in the CLAVATA signaling pathway, which is crucial for the maintenance and development of shoot apical meristems (John et al., 2023; Pan et al., 2016). In addition, the *Arabidopsis* homolog AtRLP15 is involved in both growth (Pan et al., 2016; G. D. Wang et al., 2009) and defense responses (Jones & Dangl, 2006; Y. X. Zhang et al., 2010), suggesting that FBL regulation may be linked to a broader network of plant developmental processes. Furthermore, we found that the expression of *Ghir_A10G014390* in accessions containing the CC haplotype was significantly higher than in those with the TT haplotype (*p *< 0.01) (Figure 3e). Therefore, *Ghir_A10G014390* may be a potential candidate gene influencing FBL in cotton. Additionally, *Ghir_D03G011390* is in the *qFBL‐D03‐17* region and is homologous to *REF6*. REF6 regulates multiple BR‐responsive genes and interacts with bri1‐EMS‐SUPPRESSOR 1 to mediate BR signaling (C. Li et al., 2016; Lu et al., 2011). BR influence various aspects of plant growth and development, including microtubule formation, flowering time control, and cell elongation (Li & Chory, 1999; Nolan et al., 2020). The absence of REF6 leads to reduced cell elongation, resulting in shorter leaves and petioles compared to wild‐type plants (C. Li et al., 2016). Furthermore, in *Arabidopsis*, mutations in *AtELF6* or *AtREF6* impaired cell elongation and reduced BR levels, affecting branch development (X. F. Yu et al., 2008). Additionally, the identification of candidate genes such as *Ghir_A10G014390* and *Ghir_D03G011390* further underscores the potential of these regions. The discovery that superior haplotypes, such as the CC genotype of *Ghir_A10G014390* and the GG genotype of *Ghir_D03G011390*, are associated with increased FBL and enhanced fiber quality and yield, offers new avenues for targeted breeding. These haplotypes can be selectively incorporated into breeding programs tailored to specific cotton‐growing regions, such as the Yellow River and Yangtze River valleys or the Northwest Inland Cotton Area, where traits like plant architecture and mechanical harvest suitability are critical. From the above‐mentioned results, we inferred that *Ghir_A10G014390* and *Ghir_D03G011390* were two major candidate genes that may play an important role in cotton FBL.

This study preliminarily revealed the expression differences of candidate genes *Ghir_A10G014390* and *Ghir_D03G011390* between haplotypes through small‐scale qRT‐PCR. Future studies should integrate spatiotemporal transcriptomics and metabolomics to dissect the FBL‐centered regulatory network. Additionally, CRISPR‐Cas9‐mediated gene knockout and overexpression lines could be developed to functionally validate these candidates. Combined with the field high‐throughput phenotypic platform, a genotype‐phenotypic‐environment interaction model may be established to better verify the function of candidate genes and provide targets for molecular design breeding.

The findings of this study provide valuable insights for optimizing FBL and related traits in cotton breeding. By leveraging the genetic diversity and specific haplotypes identified in this research, breeders can develop cotton varieties that are better suited to regional growing conditions and market demands, ultimately enhancing cotton production efficiency and profitability. Future research should focus on further validating these candidate genes and exploring their interaction with other traits to fully harness their potential in improving cotton cultivars.

## AUTHOR CONTRIBUTIONS

**Hui Chang**: Investigation; writing—original draft. **Honghu Ji**: Data curation; software; visualization. **Ruijie Liu**: Validation. **Juling Feng**: Investigation; visualization. **Jiayi Wang**: Validation; visualization. **Shuqi Zhao**: Investigation; validation; visualization. **Wei Li**: Investigation; validation. **Zehua Qiu**: Methodology; validation. **Nabil Ibrahim Elsheery**: Writing—original draft; writing—review and editing. **Shuxun Yu**: Conceptualization; writing—original draft; writing—review and editing. **Libei Li**: Conceptualization; data curation; funding acquisition; writing—original draft. **Zhen Feng**: Conceptualization; supervision.

## CONFLICT OF INTEREST STATEMENT

The authors declare no conflicts of interest.

## Supporting information



Supporting Information

Supporting Information

## Data Availability

All data generated or analyzed during this study are included in this published article (and its supplementary information files).
