# Supplementary material for: Genome‐wide association study reveals significant loci and candidate genes for fruit branch length in upland cotton
Source: Plant Genome. 2025 May 29;18(2):e70041. doi: 10.1002/tpg2.70041 (PMC12122414; doi:10.1002/tpg2.70041)
Supplement: Supplementary file 1 [file TPG2-18-e70041-s001.docx]

**Table S1: List of primers uesd for qRT-PCR.**

| Primer name | primer sequence（5' to 3'） |
| --- | --- |
| qGhir_A10G014390-F | AAGGAACACTTCATCTTCAAGC |
| qGhir_A10G014390-R | AGTTGTCTTCCACTTCCATAGG |
| qGhir_D03G011390-F | AAAACATCGGCCCGTTCAGA |
| qGhir_D03G011390-R | AACAATGACCGGTGTCTCCC |
| qGhActin4-F | TTGCAGACCGTATGAGCAAG |
| qGhActin4-R | ATCCTCCGATCCAGACACTG |

| **Table S2: Phenotype of FBL in the natural populations.** | | | | | | | | |
| --- | --- | --- | --- | --- | --- | --- | --- | --- |
| Line | Cultivar/Germplasm line | SRA ID | E1 | E2 | E3 | E4 | E5 | E6 |
| S1 | Zhong2191 | SRR5663653 | 17.46 | 24.41 | 18.47 | 20.56 | 23.28 | 20.98 |
| S2 | Zhong40418 | SRR5665603 | 14.69 | 19.94 | 14.47 | 18.6 | 21.09 | 17.89 |
| S3 | Zhong40618 | SRR5665624 | 13.61 | 18.26 | 12.42 | 14.36 | 15.74 | 15.04 |
| S4 | Zhong40612 | SRR5666379 | 11.67 | 15.02 | 11.81 | 12.16 | 14.37 | 13.12 |
| S5 | Zhong51811 | SRR5678372 | 11.52 | 17.09 | 12.89 | 13.56 | 10.35 | 13.07 |
| S6 | CRI94A1822 | SRR5678379 | 13.5 | 15.94 | 15.25 | 14.89 | 12.76 | 14.4 |
| S7 | Zhong61832 | SRR5690626 | 14.93 | 20.19 | 16.58 | 18.53 | 17.46 | 17.54 |
| S8 | Zhong71239 | SRR5690628 | 13.43 | 15.44 | 15.89 | 16.72 | 14.65 | 15.11 |
| S9 | PB12-1-10 | SRR5712665 | 16.8 | 17.15 | 18.25 | 21.94 | 16.76 | 17.97 |
| S10 | PB12-1-7 | SRR5712666 | 17.07 | 19.57 | 12.44 | 18.39 | 15.46 | 16.72 |
| S11 | PB12-1-8 | SRR5712668 | 17.02 | 18.33 | 12.94 | 16.17 | 15.13 | 16.07 |
| S12 | Zhong1476 | SRR5712674 | 11.09 | 14.65 | 6.72 | 10.61 | 9.94 | 10.82 |
| S13 | Zhong151222 | SRR5712676 | 13.13 | 17.33 | 9.6 | 15.69 | 11.46 | 13.53 |
| S14 | Zhong152201 | SRR5712677 | 15.06 | 21.02 | 9.11 | 13.08 | 13.76 | 14.77 |
| S15 | Zhong152201SQ | SRR5712678 | 12.87 | 20.57 | 7.81 | 14.06 | 11.81 | 13.7 |
| S16 | Zhong152214 | SRR5712679 | 14.5 | 21.69 | 10.83 | 19.25 | 20.91 | 18.11 |
| S17 | Zhong152224 | SRR5712704 | 13.3 | 16.46 | 8.5 | 16.47 | 19.83 | 15.18 |
| S18 | SS2011 | SRR5713197 | 15.35 | 14.98 | 12.89 | 14.36 | 12.74 | 14.11 |
| S19 | CQ2012-3 | SRR5713256 | 11.76 | 13.67 | 7.5 | 14.17 | 13.07 | 12.33 |
| S20 | CQ2012-4 | SRR5713307 | 12.3 | 15.44 | 11.39 | 16.5 | 19.7 | 15.19 |
| S21 | Zhong29T41 | SRR5713311 | 13.98 | 14.76 | 9.75 | 15 | 12.04 | 13.19 |
| S22 | Zhong29T42 | SRR5713312 | 16.65 | 19.2 | 11.39 | 19.97 | 16.31 | 16.82 |
| S23 | L426 | SRR5713313 | 13.85 | 16.69 | 11.61 | 16.75 | 15.26 | 14.9 |
| S24 | K640 | SRR5713314 | 14.09 | 16.67 | 13.25 | 15.55 | 17.44 | 15.51 |
| S25 | N82 | SRR5713315 | 15.92 | 16.37 | 5.61 | 12.47 | 11.67 | 12.6 |
| S26 | P21-6-7 | SRR5713316 | 24.57 | 30.37 | 14.03 | 48.03 | 37.17 | 30.81 |
| S27 | Shannong SF06 | SRR5713317 | 19.5 | 27.09 | 11.53 | 34.49 | 28.28 | 24.31 |
| S28 | sGK16 | SRR5713461 | 14.72 | 18.74 | 9.28 | 17.94 | 24.07 | 17.32 |
| S29 | V321-20-14 | SRR5713462 | 19.78 | 24.63 | 11.42 | 27 | 24.09 | 21.63 |
| S30 | Baimian 17 | SRR5713463 | 13.44 | 20.41 | 8.67 | 15.58 | 19.22 | 15.84 |
| S31 | Chaoaoyang 1 | SRR5713465 | 25.43 | 30.3 | 13.14 | 30.28 | 29.26 | 26.12 |
| S32 | Deltapine 20 | SRR5713466 | 20.3 | 23.33 | 11.03 | 25.53 | 27.7 | 21.94 |
| S33 | De 97-047 | SRR5722989 | 19.65 | 26.76 | 12.97 | 27.52 | 24.19 | 22.44 |
| S34 | Guannong 1 | SRR5723027 | 24.65 | 24.56 | 10.44 | 32.31 | 26.28 | 23.9 |
| S35 | Han2490 | SRR5723063 | 27 | 22.72 | 16.81 | 27.5 | 23.35 | 23.62 |
| S36 | Han656 | SRR5723112 | 16.22 | 14.54 | 9.36 | 12.33 | 16.54 | 14.23 |
| S37 | Han559 | SRR5723114 | 15.39 | 16.44 | 6.44 | 10.25 | 14.74 | 13.13 |
| S38 | Han667 | SRR5723117 | 17.33 | 21.07 | 9.5 | 15.75 | 16.96 | 16.51 |
| S39 | Han686 | SRR5723119 | 14.09 | 15.07 | 7.76 | 13.03 | 16.39 | 13.59 |
| S40 | Han9609 | SRR5723120 | 13.24 | 19.61 | 7.19 | 12.42 | 15.46 | 14.01 |
| S41 | Heishanmian1 | SRR5723122 | 18.35 | 25.04 | 9.33 | 21.33 | 28.8 | 21.15 |
| S42 | Jinmian3 | SRR5723124 | 23.2 | 27.19 | 10.53 | 25.56 | 26.8 | 23.17 |
| S43 | Jinmian10 | SRR5723125 | 20.28 | 20.5 | 11.78 | 28.39 | 23.59 | 21 |
| S44 | Jinmian21 | SRR5723127 | 23.26 | 27.41 | 10.81 | 34.89 | 35.63 | 26.79 |
| S45 | Jinmian23 | SRR5723128 | 21.76 | 21.46 | 10.78 | 26.26 | 24.61 | 21.25 |
| S46 | Jinmian5 | SRR6075042 | 26.22 | 25.33 | 12.36 | 29.06 | 23.31 | 23.54 |
| S47 | Liaomian10 | SRR6075043 | 22.11 | 22.52 | 18.22 | 26.31 | 27.91 | 23.54 |
| S48 | Liaomian17 | SRR6075045 | 21.81 | 23.72 | 13.97 | 24.25 | 29.2 | 22.98 |
| S49 | Liaomian5 | SRR6075047 | 20.63 | 20.61 | 11.97 | 18.17 | 24.44 | 19.62 |
| S50 | Liaomian6 | SRR6079356 | 17.96 | 24.78 | 10.67 | 21.19 | 23 | 19.92 |
| S51 | Liaomian7 | SRR6079359 | 18.09 | 20.04 | 13 | 18.19 | 21.43 | 18.43 |
| S52 | Liaomian9 | SRR6079366 | 18.02 | 24.54 | 18.31 | 22.17 | 26.11 | 22 |
| S53 | Liaoyangduanjie | SRR6079373 | 21.26 | 24.72 | 21.28 | 27.5 | 22.07 | 23.25 |
| S54 | Lu154 | SRR6079374 | 18.61 | 21.48 | 19.67 | 23.35 | 25.96 | 21.85 |
| S55 | Lumianyan19 | SRR6079383 | 14.57 | 23.13 | 15.14 | 18.25 | 22.26 | 18.89 |
| S56 | Lumian2153 | SRR6079384 | 15.57 | 21.85 | 8.64 | 16.92 | 22.11 | 17.49 |
| S57 | Nongken5 | SRR6079387 | 21.3 | 26.52 | 12.22 | 30.78 | 27.83 | 23.98 |
| S58 | Shan70 | SRR6079422 | 26.31 | 21.89 | 20.47 | 32.78 | 29.06 | 26.04 |
| S59 | Shizao1 | SRR6079423 | 14.48 | 20.26 | 17.44 | 22.28 | 25.94 | 20.11 |
| S60 | Shizao2 | SRR6079426 | 19.09 | 18.57 | 16.53 | 23.58 | 23.48 | 20.27 |
| S61 | Shizao3 | SRR6079427 | 16.11 | 20.5 | 9.39 | 13.01 | 23.11 | 17.01 |
| S62 | X13-7 | SRR6080235 | 12.13 | 16.26 | 9.14 | 19.67 | 14.89 | 14.42 |
| S63 | X25TF | SRR6080238 | 14.61 | 22.7 | 15.58 | 21.17 | 27.65 | 20.56 |
| S64 | Xiazao1 | SRR6080239 | 16.43 | 17.72 | 7.64 | 14.08 | 14.39 | 14.41 |
| S65 | Xiazao2 | SRR6080240 | 7.93 | 12.33 | 4.72 | 6.53 | 11.31 | 8.46 |
| S66 | Xiazao3 | SRR6080241 | 23.69 | 29.2 | 17.28 | 26.75 | 30.74 | 26.43 |
| S67 | Xinluzao11 | SRR6080242 | 17.63 | 24.04 | 9.03 | 23.75 | 25.69 | 20.43 |
| S68 | Xinluzao36 | SRR6080243 | 18.17 | 20.46 | 6.86 | 21.46 | 27.44 | 19.4 |
| S69 | Xinluzao3 | SRR6080244 | 13.83 | 19.43 | 4.5 | 6.44 | 8.93 | 11.2 |
| S70 | Xinluzao42 | SRR6080245 | 18.76 | 25.37 | 6.58 | 31.03 | 38.59 | 24.65 |
| S71 | Xinluzao45 | SRR6112428 | 27.48 | 34.13 | 9.28 | 35.86 | 38.07 | 29.68 |
| S72 | Xinluzao4 | SRR6112429 | 27.07 | 25.43 | 9 | 27.33 | 37 | 25.94 |
| S73 | Xinluzao6 | SRR6112430 | 18.96 | 20.52 | 13.83 | 22.6 | 32.56 | 22.08 |
| S74 | Xinluzao8 | SRR6112435 | 19.65 | 22.28 | 14.4 | 32.49 | 31.31 | 24.66 |
| S75 | Xinluzao9 | SRR6112439 | 25.13 | 32.94 | 17.56 | 30.83 | 38.81 | 29.6 |
| S76 | Mianxiang368 | SRR6112440 | 17.39 | 23.13 | 14.72 | 25.89 | 32.3 | 22.95 |
| S77 | Yu1335 | SRR6112441 | 21.74 | 30.17 | 22.47 | 31.83 | 39.37 | 29.33 |
| S78 | Yumian12 | SRR6112472 | 12.33 | 23.41 | 19.5 | 21.56 | 29.2 | 21.27 |
| S79 | Yuzao8E13 | SRR6112473 | 18.28 | 25.72 | 15.42 | 27.28 | 42.19 | 26.27 |
| S80 | Yuzao9110 | SRR6112474 | 12.48 | 17.09 | 9.67 | 15.44 | 30.3 | 17.49 |
| S81 | Jinmian57 | SRR6117358 | 13.93 | 19.52 | 10.56 | 17.25 | 23.81 | 17.36 |
| S82 | YunzaoN95 | SRR6117359 | 18.83 | 23.72 | 12.39 | 28.82 | 29.07 | 22.79 |
| S83 | Zhong416 | SRR6117360 | 14.09 | 22.15 | 11.33 | 21.06 | 29.22 | 19.95 |
| S84 | Zhong425-5 | SRR6117376 | 18.81 | 18.44 | 13.17 | 21.75 | 34.88 | 21.85 |
| S85 | Zhong716 | SRR6117377 | 20.04 | 26.91 | 11.94 | 29.08 | 40.19 | 26.2 |
| S86 | Zhong213 | SRR6117378 | 12.91 | 15.81 | 7.31 | 11.42 | 13.06 | 12.41 |
| S87 | Zhongchuang 88 | SRR6117379 | 31.57 | 27.46 | 13.53 | 37.22 | 44.54 | 31.47 |
| S88 | CRI10 | SRR6117380 | 14.91 | 19.85 | 6.44 | 14.94 | 26.76 | 17.24 |
| S89 | CRI14 | SRR6117381 | 14.2 | 20.65 | 8.19 | 14.42 | 22.61 | 16.54 |
| S90 | CRI16 | SRR6117382 | 16.19 | 24.39 | 7.56 | 19.28 | 27 | 19.49 |
| S91 | CRI20 | SRR6117384 | 19.56 | 30.91 | 12.49 | 20.58 | 36.5 | 24.84 |
| S92 | CRI24 | SRR6117385 | 22.28 | 22.28 | 7.81 | 18.11 | 23.89 | 19.61 |
| S93 | CRI27 | SRR6117386 | 16.44 | 20.13 | 9.94 | 17.69 | 27.96 | 18.95 |
| S94 | CRI30 | SRR6117387 | 13 | 18.15 | 10.28 | 15.17 | 22.02 | 16.06 |
| S95 | CRI36 | SRR6117389 | 13.5 | 28 | 4.86 | 13.39 | 20.65 | 16.85 |
| S96 | Han256 | SRR6117390 | 25.63 | 16.44 | 12.94 | 34.92 | 35.26 | 25.16 |
| S97 | CRI37 | SRR6117391 | 12.31 | 19.24 | 11 | 17.03 | 24.56 | 17.14 |
| S98 | CRI42 | SRR6117392 | 14.54 | 21.35 | 10.61 | 19.89 | 25.28 | 18.68 |
| S99 | CRI50 | SRR6117393 | 16.2 | 17.13 | 11.67 | 18.33 | 24.57 | 17.87 |
| S100 | CRI58 | SRR6117394 | 18.15 | 22.22 | 11.64 | 21.44 | 33.56 | 21.94 |
| S101 | CRI64 | SRR6117395 | 20.2 | 24 | 25.61 | 31.19 | 30.81 | 26.14 |
| S102 | CRI74 | SRR6117396 | 15.61 | 21.33 | NA | 11.06 | 23.63 | 16.85 |
| S103 | Zhong00776 | SRR6117461 | 23.43 | 22.5 | 11.64 | 20.06 | 28.87 | 21.9 |
| S104 | Zhong20398 | SRR6117401 | 22.85 | 24.57 | 12.67 | 31.75 | 35.67 | 25.87 |
| S105 | Zhong61930 | SRR6117403 | 26.81 | 29 | 16.92 | 30.11 | 35.76 | 28.19 |
| S106 | Zhong61995 | SRR6117404 | 24.98 | 28.7 | 16.61 | 27.22 | 34.31 | 26.86 |
| S107 | Zhong102909 | SRR6117438 | 24 | 33.52 | 20.67 | 30.78 | 37.56 | 29.7 |
| S108 | Zhong103026 | SRR6117440 | 26.22 | 34.81 | 19.89 | 38.44 | 34.07 | 30.86 |
| S109 | Zhong103028 | SRR6117442 | 24.24 | 30.87 | NA | 21 | 38.19 | 25.54 |
| S110 | Zhong103030 | SRR6117443 | 20.83 | 27.93 | 14.81 | 29 | 33.57 | 25.6 |
| S111 | Zhong103032 | SRR6117444 | 24.44 | 34.52 | 24.39 | 31.83 | 34.94 | 30.58 |
| S112 | Zhong103075 | SRR6117445 | 19.65 | 27.2 | 11.94 | 21.39 | 31.13 | 23.53 |
| S113 | Zhong103164 | SRR6117446 | 26.72 | 38.7 | 23.39 | 37.22 | 43.3 | 34.26 |
| S114 | Zhong103425 | SRR6117448 | 22.59 | 25.11 | 12.56 | 22.24 | 34.76 | 24.12 |
| S115 | Zhong103297 | SRR6117451 | 18.37 | 24.09 | 10.06 | 19.14 | 31.31 | 21.26 |
| S116 | Zhong109056 | SRR6117453 | 23.74 | 28.94 | 11.5 | 37.88 | 36.7 | 28.09 |
| S117 | CPB12-1-7 | SRR6117455 | 25 | 25.93 | 14.33 | 29.89 | 35.15 | 26.5 |
| S118 | CPB12-1-9 | SRR6117456 | 21.56 | 27.44 | NA | 25.09 | 33.81 | 24.06 |
| S119 | CPB12-2-7 | SRR6117458 | 22.09 | 30.44 | 13.5 | 31.33 | 42.61 | 28.62 |
| S120 | 298 | SRR6117459 | 21.54 | 31.69 | 13.14 | 36.7 | 43.93 | 29.89 |
| S121 | Zhong602186 | SRR6122540 | 22.44 | 27.06 | 14.83 | 35.11 | 34.02 | 26.88 |
| S122 | CG3020-1 | SRR6122541 | 21.43 | 30.15 | 12.58 | 31.64 | 35 | 26.61 |
| S123 | CG3020-3 | SRR6122542 | 26.11 | 22.52 | 8.51 | 32.5 | 40.7 | 26.69 |
| S124 | FM1735 | SRR6122543 | 25.56 | 27.02 | 9.97 | 46.74 | 39.85 | 29.99 |
| S125 | G2005 | SRR6122544 | 31.3 | 32.96 | 14.57 | 41 | 40.81 | 32.61 |
| S126 | Zhongzhimian2 | SRR6122545 | 24.46 | 25.57 | 13.28 | 35.17 | 36.91 | 27.4 |
| S127 | H109 | SRR6122546 | 24.2 | 27.41 | 13.69 | 35.81 | 34.5 | 27.39 |
| S128 | H559 | SRR6122547 | 27.35 | 31.15 | 22.11 | 38.25 | 44.19 | 32.88 |
| S129 | LIH33 | SRR6122548 | 28.2 | 28.52 | 16.83 | 35.46 | 38.98 | 29.98 |
| S130 | Phy-7 | SRR6122549 | 27.98 | 34.46 | 14.42 | 32.19 | 33.65 | 29.12 |
| S131 | STS458 | SRR6122550 | 28.48 | 28.3 | 16.25 | 33 | 36.35 | 28.9 |
| S132 | TM-1 | SRR6122551 | 26.02 | 28.54 | 22.64 | 37.19 | 32.81 | 29.39 |
| S133 | Aoshimian 6 | SRR6122552 | 26.96 | 25.81 | 15.53 | 47.17 | 39.41 | 30.93 |
| S134 | Baimian985 | SRR6161723 | 20.17 | 21.63 | NA | 30.75 | 28.33 | 22.54 |
| S135 | Cang198 | SRR6161724 | 19.98 | 27.89 | 10.14 | 29.83 | 33.37 | 24.72 |
| S136 | CBB | SRR6161725 | 22.41 | 25.93 | 13.39 | 26.19 | 39.98 | 26.22 |
| S137 | CNB | SRR6161726 | 21.07 | 28.24 | 13.03 | 33.47 | 38.26 | 27.21 |
| S138 | Deltapine14 | SRR6161727 | 32.5 | 33.39 | 11.11 | 30.56 | 37.58 | 29.94 |
| S139 | Deltapine15 | SRR6161728 | 28.33 | 35.37 | 10.5 | 35.97 | 38.39 | 31.61 |
| S140 | Fanmian3 | SRR6161729 | 25.57 | 31.61 | 17.94 | 30.92 | 44.46 | 30.73 |
| S141 | Ganzao109 | SRR6161730 | 27.73 | 27.87 | 16.69 | 38.58 | 37.09 | 29.81 |
| S142 | Guoxinmian11 | SRR6161731 | 27.37 | 37.19 | 14 | 33.33 | 38.31 | 31.73 |
| S143 | Han7860 | SRR6161733 | 29.56 | 27.74 | 11.58 | 31.94 | 32.74 | 27.26 |
| S144 | Ji4025 | SRR6161734 | 26.04 | 34.44 | 15.17 | 38.25 | 35.78 | 30.29 |
| S145 | Jimian 26 | SRR6161735 | 18.24 | 20.67 | 17.14 | 30.25 | 38.76 | 25.16 |
| S146 | BM03 | SRR6161736 | 24.19 | 30.91 | 14.28 | 30.17 | 38.83 | 28.28 |
| S147 | Kelin098 | SRR6161737 | 26.07 | 31.44 | 12.64 | 31.82 | 37.19 | 28.45 |
| S148 | Liaomian23 | SRR6161738 | 27.15 | 25.83 | 7.22 | 29.14 | 38.48 | 26.39 |
| S149 | Liaomian27 | SRR6161790 | 22.91 | 25.2 | 11.11 | 24.53 | 40.81 | 25.7 |
| S150 | Liaomian28 | SRR6161791 | 23.96 | 32.76 | 13.72 | 30.17 | 40.7 | 28.97 |
| S151 | Lu05R59 | SRR6164732 | 25.19 | 30.13 | 13.78 | 42.14 | 39.83 | 30.46 |
| S152 | Lu7619 | SRR6164733 | 23.41 | 30.74 | 10.58 | 30.11 | 37.96 | 27.25 |
| S153 | Lumianyan17 | SRR6164734 | 24.15 | 30.19 | 14.72 | 38.75 | 46 | 31.21 |
| S154 | Lumianyan21 | SRR6164735 | 21.8 | 29.31 | 15.44 | 33.64 | 39.74 | 28.37 |
| S155 | Lumianyan28 | SRR6164795 | 25.78 | 30 | 10.06 | 26.97 | 42.04 | 27.91 |
| S156 | Lumianyan36 | SRR6164796 | 20.65 | 26.41 | 8.53 | 19.92 | 36.19 | 23.24 |
| S157 | Lumianyan38 | SRR6165238 | 23.59 | 29.54 | 10.2 | 29.42 | 39.61 | 27.21 |
| S158 | Miaobao21 | SRR6165239 | 23.43 | 27.91 | 13.25 | 31.83 | 40.36 | 27.89 |
| S159 | Renhe39 | SRR6165240 | 25.87 | 35.39 | 17.33 | 28.39 | 42.96 | 30.78 |
| S160 | Rihuimian6 | SRR6165241 | 22.74 | 33.43 | 15.28 | 30.53 | 41.41 | 29.32 |
| S161 | ShannongSF01 | SRR6165242 | 27.81 | 37.54 | 18.42 | 45.67 | 45.22 | 35.25 |
| S162 | Shan79 | SRR6165243 | 27.28 | 34.5 | 20.19 | 35.93 | 43.43 | 32.73 |
| S163 | Stoneville 2B | SRR6165260 | 27.07 | 35.94 | 19.33 | 32.92 | 41.83 | 32.01 |
| S164 | Xinmian 33B | SRR6165261 | 25.61 | 38.13 | 20.28 | 35.25 | 42.76 | 32.92 |
| S165 | Xinzhimian5 | SRR6173347 | 22.63 | 25.78 | 17.17 | 24.08 | 41.2 | 26.79 |
| S166 | Yinhuashu | SRR6173943 | 26.54 | 29.8 | 18.75 | 34.93 | 38.22 | 29.96 |
| S167 | You009 | SRR6173944 | 25.72 | 32.17 | NA | 41 | 37.41 | 30.3 |
| S168 | Zhong109 | SRR6173945 | 22.81 | 34.93 | 16.83 | 39.75 | 39.09 | 30.95 |
| S169 | CRI17 | SRR6173946 | 22.06 | 27.5 | 14.69 | 25.89 | 35.04 | 25.56 |
| S170 | CRI19 | SRR6174441 | 22.69 | 30.48 | 19.92 | 32.14 | 34.69 | 28.2 |
| S171 | CRI43 | SRR6174442 | 23.57 | 29.17 | 17.72 | 35.33 | 39.3 | 29.3 |
| S172 | CRI60 | SRR6174443 | 20.89 | 27.07 | 12.58 | 18.36 | 33.56 | 23.27 |
| S173 | Zhong662 | SRR6174444 | 12.52 | 24.3 | 6.64 | 15.47 | 33.78 | 19.37 |
| S174 | Zhong679 | SRR6174445 | 24.41 | 30.46 | 9.25 | 46.47 | 37.69 | 29.85 |
| S175 | Zhong69 | SRR6174446 | 29.44 | 32.19 | 12.69 | 43.08 | 35.8 | 30.95 |
| S176 | Zhong800319 | SRR6174447 | 29.2 | 36.37 | 12.42 | 55.45 | 42.83 | 35.4 |
| S177 | CRI 94A915 | SRR6174448 | 20.96 | 23.54 | 11.72 | 41.03 | 38.52 | 27.24 |
| S178 | CRI12 | SRR6174455 | 21.39 | 29.5 | 10.44 | 34.97 | 37.26 | 27.16 |
| S179 | CRI35 | SRR6174456 | 23.37 | 28.19 | 15.31 | 32.36 | 35.28 | 27.24 |
| S180 | CRI41 | SRR6174457 | 25.24 | 27.11 | 11.81 | 40.57 | 40.94 | 29.46 |
| S181 | CRI45 | SRR6174458 | 21.96 | 25.65 | 14.25 | 29.81 | 36.48 | 26.03 |
| S182 | CRI49 | SRR6174459 | 17.46 | 24.76 | 11.22 | 19.78 | 31.35 | 21.62 |
| S183 | CRI7 | SRR6174460 | 22.24 | 25.26 | 11.11 | 22.44 | 34.22 | 23.75 |
| S184 | Zhongzhimian 8 | SRR6174461 | 22.89 | 28.28 | 12.36 | 30.92 | 35.41 | 26.45 |
| S185 | Zhongzhimian GD89 | SRR6174462 | 21.7 | 26.06 | 13.58 | 21.17 | 31.89 | 23.49 |
| S186 | Shan920346 | SRR6174463 | 22.33 | 31.35 | 14.39 | 31.53 | 39.56 | 28.37 |
| S187 | XianⅢ9704 | SRR6174464 | 12.44 | 12.92 | 6.44 | 12.19 | 14.3 | 11.78 |
| S188 | US-1 | SRR6174465 | 28.74 | 32.04 | 14.11 | 42.36 | 43.3 | 32.54 |
| S189 | Kyrgyzstan cotton | SRR6174466 | 21.24 | 25.04 | 4.81 | 20.86 | 25.7 | 20.27 |
| S190 | Bazhou5409 | SRR6174467 | 15.41 | 20.41 | 8.17 | 17.56 | 16.65 | 15.95 |
| S191 | Yumian1 | SRR6174468 | 14.33 | 19.31 | 9.17 | 12.11 | 18.76 | 15.19 |
| S192 | Huazhong910102 | SRR6174469 | 27.11 | 32.93 | 12.94 | 34.94 | 38.63 | 29.91 |
| S193 | Israel cotton | SRR6174470 | 21.5 | 36.09 | 15.56 | 22.86 | 34.94 | 26.97 |
| S194 | Ken6614 | SRR6174471 | 19.65 | 29.93 | 5.22 | 22.75 | 25.57 | 21.36 |
| S195 | Ken0074 | SRR6174472 | 22.3 | 31.89 | 6.67 | 21.77 | 26.48 | 22.67 |
| S196 | Chuan239-1 | SRR6174473 | 22.76 | 28.31 | 8.44 | 31.31 | 36.37 | 26.06 |
| S197 | Bamian3 | SRR6174474 | 22.26 | 32.19 | 11.61 | 25.56 | 39.59 | 27.09 |
| S198 | Chuan338 | SRR6174595 | 22.65 | 34.78 | 14.67 | 30.86 | 42.39 | 29.77 |
| S199 | Chuan267 | SRR6174596 | 23.85 | 32.94 | 12 | 31.94 | 30.33 | 26.69 |
| S200 | Chuan65 | SRR6174597 | 29.46 | 41.13 | 15.5 | 40.31 | 45.11 | 35.01 |
| S201 | Chuanjian1 | SRR6174598 | 23.83 | 32.39 | 10.72 | 33.17 | 45.72 | 29.97 |
| S202 | Xinluzao 2 | SRR6174599 | 23.43 | 25.52 | 7.44 | 25.67 | 44.02 | 26.18 |
| S203 | Xinluzao10 | SRR6174600 | 19.98 | 26.15 | 6.5 | 23.03 | 28.46 | 21.5 |
| S204 | Xinluzao12 | SRR6174601 | 32.8 | 34.33 | 20.17 | 47.33 | 57.04 | 38.84 |
| S205 | Xinluzao13 | SRR6174602 | 20.74 | 31.61 | 14.33 | 29.19 | 47.43 | 29.43 |
| S206 | Xinluzao15 | SRR6174603 | 28.06 | 30.81 | 15.5 | 31.42 | 39.81 | 29.75 |
| S207 | Xinluzao16 | SRR6174604 | 18.59 | 26.04 | 9.67 | 21.53 | 40.19 | 24.05 |
| S208 | Xinluzao17 | SRR6174605 | 17.69 | 40.17 | 12.94 | 22.89 | 37 | 27.05 |
| S209 | Xinluzao18 | SRR6174606 | 23.31 | 22.13 | 8.06 | 25.11 | 26.39 | 21.49 |
| S210 | Xinluzao19 | SRR6174667 | 21.7 | 28.13 | 11.11 | 24.11 | 35.81 | 24.9 |
| S211 | Xinluzao20 | SRR6174668 | 18.94 | 24.43 | 14.93 | 24.03 | 29.09 | 22.6 |
| S212 | Xinluzao21 | SRR6174669 | 24.39 | 23.65 | 13.17 | 37.64 | 43.56 | 28.82 |
| S213 | Xinluzao22 | SRR6174670 | 21.2 | 24.7 | 9.67 | 26.06 | 42.2 | 25.53 |
| S214 | Xinluzao23 | SRR6174671 | 23.98 | 33.31 | 17.13 | 36.72 | 48.61 | 32.51 |
| S215 | Xinluzao24 | SRR6174672 | 18.35 | 28.87 | 8.33 | 24.56 | 39.35 | 24.72 |
| S216 | Yumian5 | SRR6174673 | 22.69 | 30.04 | 7.94 | 29.86 | 39.39 | 26.77 |
| S217 | Yumian18 | SRR6174674 | 25.06 | 35.35 | 8.33 | 31.56 | 42.62 | 29.54 |
| S218 | Yumian21 | SRR6174675 | 22.44 | 28.96 | 13.72 | 33.47 | 36.33 | 27.36 |
| S219 | Yun1729 | SRR6174676 | 24.22 | 29.24 | 23.5 | 43.61 | 39.98 | 31.26 |
| S220 | Zhemian11 | SRR6174677 | 19.8 | 27.44 | 28.11 | 30.19 | 38.13 | 28.69 |
| S221 | Tkuo | SRR6174678 | 21.81 | 30.81 | 15.67 | 27.68 | 36.83 | 27.11 |
| S222 | Bo425 | SRR6174679 | 26.15 | 34.06 | 6.17 | 31.58 | 31.52 | 26.67 |
| S223 | Xinluzhong 60 | SRR6174680 | 29.5 | 25.87 | 7.17 | 23.64 | 32.44 | 24.65 |
| S224 | Ken27-3 | SRR6174681 | 16.67 | 20.11 | 8.33 | 24.32 | 20.68 | 18.21 |
| S225 | B-3 | SRR6174682 | 24.19 | 38.15 | 10.67 | 30.82 | 40.48 | 29.76 |
| S226 | Xinluzao25 | SRR6174683 | 25.04 | 29.81 | 10.11 | 26 | 36.78 | 26.38 |
| S227 | Xinluzao26 | SRR6174684 | 16.7 | 26.76 | 12.33 | 26.78 | 29.19 | 22.66 |
| S228 | Xinluzao27 | SRR6174685 | 16.67 | 27.11 | 9.78 | 22.06 | 28.65 | 21.68 |
| S229 | Xinluzao28 | SRR6174686 | 16.15 | 22.63 | 8.56 | 15.08 | 28.69 | 19.16 |
| S230 | Xinluzao29 | SRR6174687 | 23.13 | 28.81 | 7.94 | 25.44 | 32.37 | 24.3 |
| S231 | Xinluzao30 | SRR6174688 | 19.74 | 24.85 | 13.56 | 24.67 | 28.39 | 22.59 |
| S232 | Xinluzao32 | SRR6174690 | 22.81 | 23.74 | 6.89 | 32.53 | 28.06 | 23.15 |
| S233 | Xinluzao33 | SRR6174689 | 17.52 | 27.07 | 9.11 | 18.11 | 34.98 | 22.22 |
| S234 | Xinluzao34 | SRR6174691 | 21.85 | 27.39 | 7.89 | 23.59 | 32.37 | 23.38 |
| S235 | Xinluzao35 | SRR6174692 | 23.02 | 27.26 | 11.94 | 28.06 | 31.15 | 24.76 |
| S236 | Xinluzao37 | SRR6174693 | 18.02 | 24.87 | 8.5 | 22.03 | 34.83 | 22.36 |
| S237 | Xinluzao38 | SRR6174694 | 23.41 | 25 | 18.78 | 40.36 | 44.67 | 30.54 |
| S238 | Xinluzao39 | SRR6174695 | 25.89 | 32.63 | 9.22 | 30.89 | 28.56 | 26.03 |
| S239 | Xinluzao40 | SRR6174696 | 18.72 | 24.65 | 8.72 | 21.36 | 33.39 | 22.07 |
| S240 | Xinluzao41 | SRR6174697 | 27.11 | 26.8 | 11.22 | 40.47 | 48.41 | 31.35 |
| S241 | Xinluzao46 | SRR6517995 | 27.59 | 24.48 | 7.89 | 31.11 | 40.02 | 26.97 |
| S242 | Xinluzao47 | SRR6517996 | 29.93 | 28.52 | 8.67 | 36.58 | 38.96 | 29.19 |
| S243 | Xinluzao48 | SRR6517997 | 18.19 | 22.31 | 6.17 | 25.9 | 23.5 | 19.57 |
| S244 | Xinluzao49 | SRR6517998 | 28.76 | 38.06 | 11.94 | 42.64 | 49.37 | 34.92 |
| S245 | Xinluzao50 | SRR6518002 | 17.52 | 19.61 | 8.33 | 24.03 | 33.72 | 21.14 |
| S246 | Xinluzao51 | SRR6518003 | 23.85 | 23.26 | 6.39 | 22.86 | 31.61 | 22.37 |
| S247 | Xinluzhong1 | SRR6518284 | 19.61 | 24.98 | 6 | 28.17 | 32.5 | 22.83 |
| S248 | Xinluzhong3 | SRR6518285 | 17.11 | 23.26 | 7.22 | 19 | 30.35 | 20.09 |
| S249 | Xinluzhong4 | SRR6518286 | 22.06 | 27.48 | 8 | 20.33 | 28.3 | 22.02 |
| S250 | Xinluzhong5 | SRR6518288 | 33.63 | 40.87 | 12.78 | 33.44 | 32.72 | 31.53 |
| S251 | Xinluzhong6 | SRR6518290 | 22.74 | 30.85 | 10.78 | 20.97 | 37.43 | 25.52 |
| S252 | Xinluzhong7 | SRR6518297 | 21.09 | 28.98 | 10.17 | 21.78 | 29.06 | 22.91 |
| S253 | Xinluzhong8 | SRR6518298 | 24.61 | 29.07 | 9.78 | 34.44 | 38.37 | 27.83 |
| S254 | Xinluzhong9 | SRR6518300 | 26.17 | 32.19 | 10.28 | 32.5 | 47.11 | 30.57 |
| S255 | Xinluzhong10 | SRR6518348 | 21.15 | 25.39 | 5.67 | 16.86 | 28.09 | 20.34 |
| S256 | Xinluzhong12 | SRR6518349 | 28.15 | 31.28 | 10.28 | 23.15 | 35.5 | 26.67 |
| S257 | Xinluzhong13 | SRR6518350 | 19.8 | 26.57 | 11.17 | 27.25 | 39.43 | 25.47 |
| S258 | Xinluzhong14 | SRR6518352 | 24.94 | 27.94 | 11.67 | 24.56 | 39.96 | 26.67 |
| S259 | Xinluzhong15 | SRR6518371 | 22.26 | 31.54 | 10.28 | 27.69 | 32.78 | 25.57 |
| S260 | Xinluzhong16 | SRR6518372 | 25.56 | 39.85 | 19 | 39.83 | 42.17 | 33.71 |
| S261 | Xinluzhong17 | SRR6518391 | 21.61 | 30.93 | 19.56 | 34.86 | 38.67 | 29.34 |
| S262 | Xinluzhong19 | SRR6518392 | 31.41 | 36.54 | 27.67 | 36.93 | 47 | 36.31 |
| S263 | Xinluzhong20 | SRR6518393 | 21.7 | 24 | 9.78 | 30.14 | 35.41 | 24.68 |
| S264 | Xinluzhong21 | SRR6518394 | 21.74 | 28.26 | 7.78 | 26.53 | 40.22 | 25.77 |
| S265 | Xinluzhong22 | SRR6518395 | 23.81 | 30.61 | 15.17 | 27.58 | 29.65 | 25.81 |
| S266 | Xinluzhong26 | SRR6518396 | 23.59 | 30.72 | 13.5 | 37.94 | 43.87 | 30.39 |
| S267 | Xinluzhong27 | SRR6518397 | 16.26 | 18.07 | 9.39 | 22.42 | 29.81 | 19.56 |
| S268 | Xinluzhong28 | SRR6518398 | 25.11 | 27.13 | 20.5 | 44.11 | 41.52 | 31.6 |
| S269 | Xinluzhong30 | SRR6518399 | 22.43 | 26.54 | 10.83 | 26.58 | 37.69 | 25.49 |
| S270 | Xinluzhong32 | SRR6518400 | 25.24 | 26.61 | 25.28 | 35.75 | 34.31 | 29.32 |
| S271 | Xinluzhong34 | SRR6518405 | 21.24 | 23.5 | 13.17 | 34.19 | 35.61 | 25.75 |
| S272 | Xinluzhong35 | SRR6518406 | 21.63 | 24.76 | 19.89 | 29.5 | 40.76 | 27.6 |
| S273 | Xinluzhong40 | SRR6518407 | 25.22 | 26.89 | 22.06 | 32.61 | 33.09 | 28.05 |
| S274 | Xinluzhong41 | SRR6518410 | 25.24 | 38.54 | 12.89 | 38.53 | 46.35 | 33.04 |
| S275 | Xinluzhong45 | SRR6518412 | 23.85 | 30.96 | 11.28 | 39.22 | 41.07 | 29.73 |
| S276 | Xinluzhong46 | SRR6518415 | 25.56 | 30.43 | 14.28 | 44.17 | 42.85 | 31.7 |
| S277 | Xinluzhong47 | SRR6518416 | 28.37 | 29.31 | NA | 41.22 | 42.61 | 36.34 |
| S278 | Kangcaoganlin | SRR6518417 | 17.72 | 21.96 | 22.11 | 33.25 | 40.63 | 27.07 |
| S279 | Jiangyin1 | SRR6518420 | 21.46 | 28.15 | 20.5 | 33.4 | 43.04 | 29.57 |
| S280 | Huihe36 | SRR6518421 | 21.09 | 37 | 19.33 | 35.11 | 47.61 | 32.56 |
| S281 | Jinken1042 | SRR6518422 | 21.11 | 28.02 | 11.83 | 25.76 | 44.91 | 27.16 |
| S282 | Guoxinmian9 | SRR6518423 | 26.57 | 34.69 | 14.89 | 33.61 | 47.54 | 32.26 |
| S283 | Junmian1 | SRR6518424 | 28.13 | 28.67 | 9.89 | 31.19 | 41.57 | 28.71 |
| S284 | Huiyuan717 | SRR6518425 | 30.94 | 22.78 | 17.94 | 33.83 | 52.72 | 32.28 |
| S285 | Yunzao219 | SRR6518426 | 26.76 | 30.89 | 13.22 | 33.36 | 43.22 | 30.18 |
| S286 | Yunzao33-356 | SRR6518427 | 25.48 | 28.81 | 10.83 | 26.67 | 39.2 | 27.03 |
| S287 | Jinmian2 | SRR6518428 | 22.06 | 29.74 | 10.94 | 24.5 | 38.06 | 25.87 |
| S288 | Chaoyangmian2 | SRR6518429 | 19 | 31 | 4.06 | 25.47 | 27.2 | 22.08 |
| S289 | Dunhuang77-116 | SRR6518430 | 21.54 | 29.96 | 15.61 | 31.81 | 42.44 | 28.78 |
| S290 | Ganmian4 | SRR6518444 | 21.69 | 31.24 | 11.5 | 29.25 | 41.06 | 27.68 |
| S291 | Guannongzao C-50 | SRR6518445 | 24.2 | 29.8 | 11.44 | 25.69 | 33.83 | 25.71 |
| S292 | Guannongchangzao B14 | SRR6518446 | 23.35 | 28.78 | 7.67 | 31.06 | 41.54 | 27.27 |
| S293 | Yanzao1 | SRR6518447 | 16.96 | 25.52 | 10.78 | 20.89 | 30.7 | 21.54 |
| S294 | Yanzao2 | SRR6518448 | 14.67 | 24.61 | 20.17 | 18.22 | 31.87 | 22.21 |
| S295 | Jinmian6 | SRR6518449 | 19.48 | 31.11 | 10 | 23.75 | 39.61 | 25.67 |
| S296 | Jinken69-2 | SRR6518450 | 18.89 | 25.59 | 6.94 | 17.28 | 29.09 | 20.39 |
| S297 | Jinken148-39 | SRR6518451 | 22.48 | 26.24 | 10.17 | 28.47 | 38.78 | 25.88 |
| S298 | Zhuangjiahan102 | SRR6518452 | 21.13 | 28.72 | 14.72 | 24.31 | 26.19 | 23.4 |
| S299 | Yinshan4 | SRR6518453 | 23.91 | 20.11 | 7.44 | 23.58 | 24.57 | 20.41 |
| S300 | Ejing1 | SRR6518454 | 26.76 | 31.37 | 8.11 | 29.89 | 37.48 | 27.58 |
| S301 | Simian3 | SRR6518455 | 19.61 | 24.63 | 12.56 | 37.83 | 34.11 | 25.81 |
| S302 | Stoneville 4B | SRR6519349 | 21.98 | 28.96 | 8.39 | 24.9 | 32.87 | 24.17 |
| S303 | Jimian10 | SRR6519350 | 28.44 | 27.2 | 10.17 | 34.89 | 30.57 | 26.67 |
| S304 | Jimian11 | SRR6519214 | 23.33 | 26.43 | 9.11 | 28.61 | 34.15 | 24.93 |
| S305 | Jimian12 | SRR6519215 | 25.39 | 37.28 | 13.78 | 35.22 | 40.35 | 31.06 |
| S306 | Jimian16 | SRR6519216 | 29.26 | 31.13 | 15.22 | 37.19 | 40.67 | 31.19 |
| S307 | Jimian17 | SRR6519217 | 24.19 | 29.5 | 8.67 | 24.61 | 32.35 | 24.67 |
| S308 | Sumian4 | SRR6519221 | 25.44 | 24.91 | 11.56 | 33.06 | 29.65 | 25.21 |
| S309 | Ekangmian2 | SRR6519421 | 16.91 | 28.5 | 15.44 | 28.36 | 38.61 | 25.97 |
| S310 | Ekangmian3 | SRR6519422 | 25.48 | 28.11 | 16.78 | 28.39 | 37.37 | 27.74 |
| S311 | Ekangmian6 | SRR6519423 | 24.8 | 27.41 | 10.33 | 31.08 | 35.65 | 26.43 |
| S312 | Edaimian | SRR6519424 | 30.74 | 36.15 | 13.89 | 41.2 | 39.89 | 32.91 |
| S313 | Xuzhou142 | SRR6519222 | 27.2 | 31.31 | 15.39 | 35.47 | 41.67 | 30.74 |
| S314 | Sumian9 | SRR6519223 | 25.44 | 33.06 | 21.89 | 38.06 | 45.57 | 33.12 |
| S315 | Sumian12 | SRR6519224 | 22.76 | 28.89 | 13.93 | 31.36 | 32.63 | 26.28 |
| S316 | Sukang191 | SRR6519225 | 16.93 | 23.33 | 11.17 | 26.83 | 33.93 | 22.82 |
| S317 | Gangmian1 | SRR6519226 | 17.03 | 21.83 | 8.72 | 15.14 | 21.76 | 17.47 |
| S318 | Gangmian2 | SRR6519227 | 29.06 | 29.83 | 21.5 | 31.78 | 39.2 | 30.68 |
| S319 | Coker 201 | SRR6519228 | 18.87 | 29.81 | 15.06 | 37 | 39.17 | 28.2 |
| S320 | Daihongdai | SRR6519229 | 26.48 | 30.46 | 18.11 | 39.53 | 40.22 | 31.2 |
| S321 | Yishuhong | SRR6519230 | 18.17 | 18.52 | NA | 10.01 | 21.04 | 14.9 |
| S322 | Bomian1 | SRR6519231 | 26.91 | 32.74 | 24.39 | 34.75 | 43.96 | 32.88 |
| S323 | Kemian4 | SRR6519232 | 24.26 | 31.11 | 11.44 | 34.19 | 37.04 | 28.14 |
| S324 | Ganmian11 | SRR6519233 | 25.73 | 32.87 | 9.78 | 44.94 | 32.83 | 29.44 |
| S325 | Ganmian12 | SRR6519234 | 28.91 | 35.78 | 7.67 | 53.19 | 37.24 | 32.79 |
| S326 | Xianmian13 | SRR6519235 | 24.2 | 28.22 | 7.86 | 36.58 | 39.09 | 27.74 |
| S327 | Yapengmian | SRR6519236 | 20.33 | 35.13 | 10.89 | 21.32 | 23.61 | 22.94 |
| S328 | Jijiaodezimian | SRR6519237 | 21.59 | 32.78 | 10.78 | 27.78 | 32.39 | 25.71 |
| S329 | 611bo | SRR6519238 | 26.28 | 25.57 | 14.56 | 32.92 | 39.69 | 28.25 |
| S330 | Annong121 | SRR6519239 | 28.78 | 31.02 | 17.17 | 34.5 | 37.15 | 30.15 |
| S331 | Deltapine16 | SRR6519240 | 17.47 | 29.44 | 5.89 | 13.11 | 32.59 | 20.83 |
| S332 | Delfos 531 | SRR6519241 | 18.91 | 25.7 | 5.67 | 15.86 | 33.59 | 20.97 |
| S333 | Dunmian1 | SRR6519242 | 24.65 | 32.43 | 10.89 | 32.53 | 40.17 | 28.85 |
| S334 | Dunmian2 | SRR6519243 | 15.8 | 26.33 | 6.28 | 17.06 | 23.44 | 18.46 |
| S335 | Ganmian2 | SRR6519244 | 12.67 | 22.8 | 9.39 | 13.14 | 25.83 | 17.38 |
| S336 | Ganmina3 | SRR6519245 | 26.11 | 31.83 | 14.19 | 24.93 | 42.85 | 28.92 |
| S337 | Ganmian47 | SRR6519246 | 32.11 | 40 | 15.56 | 42.53 | 38.96 | 34.36 |
| S338 | Guangyedaizimian | SRR6519247 | 23.8 | 26 | 8.11 | 20.28 | 30.7 | 22.62 |
| S339 | Ji668 | SRR6519248 | 22.17 | 28.65 | 17.11 | 26.44 | 37.46 | 26.88 |
| S340 | Jimian25 | SRR6519249 | 25.69 | 28.72 | 13.06 | 22.86 | 39.56 | 26.87 |
| S341 | Jinmian5 | SRR6519250 | 20.28 | 26.5 | 17.17 | 25.57 | 34.48 | 25.47 |
| S342 | Keke1543 | SRR6519251 | 26.17 | 36.98 | 17.5 | 32.43 | 42.7 | 31.84 |
| S343 | Ningmian1 | SRR6519252 | 36.11 | 38.7 | 13.5 | 43.2 | 42.78 | 35.58 |
| S344 | Ningmian22 | SRR6519265 | 25.82 | 30.26 | 12.22 | 37.22 | 40.35 | 29.67 |
| S345 | Nongda94-7 | SRR6519266 | 25.43 | 25.96 | 8.61 | 26.59 | 39.87 | 26.15 |
| S346 | Nongdamian8 | SRR6519267 | 26.15 | 30.35 | 11.72 | 31.92 | 38.41 | 28.36 |
| S347 | Shumian1 | SRR6519268 | 27.54 | 32.39 | 13.78 | 31.81 | 38.02 | 29.36 |
| S348 | Sumian1 | SRR6519341 | 21.85 | 31.06 | 9.5 | 26.67 | 40.13 | 26.7 |
| S349 | Sumian22 | SRR6519342 | 24.57 | 31.06 | 11.78 | 25.14 | 39.31 | 27.25 |
| S350 | Xiangmian10 | SRR6519343 | 20.57 | 28.3 | 7.67 | 20.19 | 32.07 | 22.63 |
| S351 | Xuzhou219 | SRR6519344 | 25.35 | 24.94 | 9.94 | 22.53 | 33.63 | 24.06 |
| S352 | Yinshan8 | SRR6519345 | 25.23 | 28.59 | 11.28 | 36.92 | 41.37 | 29.19 |
| S353 | Yumian1 | SRR6519346 | 25.43 | 29.17 | 14.94 | 39.44 | 43.19 | 30.79 |
| S354 | Yumian2 | SRR6519347 | 28.44 | 31.43 | 10.11 | 42.19 | 42.74 | 31.52 |
| S355 | Xinluzao53 | SRR6519348 | 24.19 | 26.78 | 8.53 | 28.36 | 34.4 | 25.12 |
| E1: Liaocheng-2021; E2: Huanggang-2021; E3: Sanya-2021-2022;  E4: Liaocheng-2022; E5: Huanggang-2022; E6: Sanya-2022-2023. | | | | | | | | |

| **Table S3: Phenotypic variation of FBL in the natural populations.** | | | | | | |  |  |  |  |
| --- | --- | --- | --- | --- | --- | --- | --- | --- | --- | --- |
| Trait | Environment | Mean | Min | Max | SD | Skewness | Kurtosis | CV | *H²* |  |
|  |  |  |  |  |  |  |  |  |  |  |
| FBL | E1 | 21.76 | 7.93 | 36.11 | 4.88 | -0.18 | -0.41 | 22.43% | 65.25% |  |
|  | E2 | 26.86 | 12.33 | 41.13 | 5.70 | -0.10 | -0.25 | 21.22% |  |  |
|  | E3 | 12.55 | 4.06 | 28.11 | 4.38 | 0.76 | 0.52 | 34.90% |  |  |
|  | E4 | 27.65 | 6.44 | 55.45 | 8.72 | 0.10 | -0.31 | 31.53% |  |  |
|  | E5 | 33.08 | 8.93 | 57.04 | 8.97 | -0.63 | -0.12 | 27.00% |  |  |
|  | E6 | 24.87 | 8.46 | 38.84 | 5.58 | -0.45 | -0.60 | 22.44% |  |  |
| E1: Liaocheng-2021; E2: Huanggang-2021; E3: Sanya-2021-2022;  E4: Liaocheng-2022; E5: Huanggang-2022; E6: Sanya-2022-2023.  SD: Standard Deviation;  CV: Coefficient of Variation;  *H²*: Broad-sense heritability | | | | | | | | | |  |

| **Table S4: Variance analysis for FBL in the natural population.** | | | | | | |
| --- | --- | --- | --- | --- | --- | --- |
| Trait | Variables | *df* | Sum of squares | *F*-value | *P*-value |  |
| FBL | Environment | 5 | 238292 | 2083.07 | <0.001 | *** |
|  | Genotype | 354 | 141998 | 17.53 | <0.001 | *** |
|  | Genotype × environment | 1770 | 76311 | 1.88 | < 0.001 | *** |
|  | Error | 1052 | 24069 |  |  |  |

| **Table S5: Significant quantitative trait locus (QTLs) associated with FBL.** | | | | | |  |
| --- | --- | --- | --- | --- | --- | --- |
| ID | Chr | Position | Significant SNP | Environment | -LOG_10_(*P*) | Phenotypic variation explained |
| qFBL-A01-1 | A01 | 80069526 | rsA01_80069526 | E4 | 6.85 | 7.51% |
| qFBL-A01-2 | A01 | 100611152 | rsA01_100611152 | E1 | 5.24 | 5.67% |
| qFBL-A01-3 | A01 | 117519224 | rsA01_117519224 | E2 | 6.02 | 6.58% |
| qFBL-A02-1 | A02 | 681347 | rsA02_681347 | E1 | 5.17 | 5.57% |
|  |  | 681861 | rsA02_681861 | E1 | 5.30 | 5.66% |
|  |  | 682314 | rsA02_682314 | E1 | 5.00 | 5.38% |
| qFBL-A02-2 | A02 | 74823215 | rsA02_74823215 | E5 | 5.44 | 5.89% |
| qFBL-A03-1 | A03 | 1022534 | rsA03_1022534 | E2 | 5.04 | 5.42% |
|  |  | 1040829 | rsA03_1040829 | E2 | 5.04 | 5.42% |
|  |  | 1179754 | rsA03_1179754 | E4/E6/BLUP | 5.42 | 5.86% |
| qFBL-A03-1 | A03 | 1564814 | rsA03_1564814 | E4 | 5.16 | 5.53% |
| qFBL-A03-2 | A03 | 1564818 | rsA03_1564818 | E4 | 5.16 | 5.53% |
|  |  | 1569721 | rsA03_1569721 | E4 | 5.42 | 5.89% |
|  |  | 1582194 | rsA03_1582194 | E4 | 5.03 | 5.39% |
|  |  | 1583098 | rsA03_1583098 | E4 | 5.03 | 5.39% |
|  |  | 1584739 | rsA03_1584739 | E4 | 5.03 | 5.39% |
|  |  | 1585865 | rsA03_1585865 | E4 | 5.03 | 5.39% |
|  |  | 1592336 | rsA03_1592336 | E4 | 5.03 | 5.39% |
|  |  | 1592654 | rsA03_1592654 | E4 | 5.03 | 5.39% |
|  |  | 1600535 | rsA03_1600535 | E4 | 5.42 | 5.83% |
|  |  | 1603537 | rsA03_1603537 | E4 | 5.03 | 5.39% |
|  |  | 1614006 | rsA03_1614006 | E4 | 5.01 | 5.36% |
|  |  | 1614548 | rsA03_1614548 | E4 | 5.08 | 5.41% |
|  |  | 1615763 | rsA03_1615763 | E4 | 5.03 | 5.39% |
|  |  | 1616270 | rsA03_1616270 | E4 | 5.82 | 6.37% |
|  |  | 1620334 | rsA03_1620334 | E4 | 5.03 | 5.39% |
|  |  | 1620739 | rsA03_1620739 | E4 | 5.03 | 5.39% |
|  |  | 1620845 | rsA03_1620845 | E4 | 5.03 | 5.39% |
|  |  | 1632428 | rsA03_1632428 | E4 | 5.03 | 5.39% |
|  |  | 1632742 | rsA03_1632742 | E4 | 5.57 | 6.03% |
|  |  | 1632894 | rsA03_1632894 | E4 | 5.82 | 6.37% |
|  |  | 1633335 | rsA03_1633335 | E4/E6/BLUP | 5.71 | 6.20% |
|  |  | 1633967 | rsA03_1633967 | E4 | 5.03 | 5.39% |
|  |  | 1635899 | rsA03_1635899 | E4 | 5.03 | 5.39% |
|  |  | 1637652 | rsA03_1637652 | E4 | 5.03 | 5.39% |
|  |  | 1642749 | rsA03_1642749 | E4 | 5.18 | 5.57% |
|  |  | 1642948 | rsA03_1642948 | E4 | 5.03 | 5.39% |
|  |  | 1643004 | rsA03_1643004 | E4 | 5.03 | 5.39% |
|  |  | 1647478 | rsA03_1647478 | E4/E6/BLUP | 6.89 | 7.62% |
|  |  | 1647740 | rsA03_1647740 | E4 | 5.06 | 5.39% |
|  |  | 1650971 | rsA03_1650971 | E4 | 5.03 | 5.39% |
|  |  | 1651843 | rsA03_1651843 | E4 | 5.19 | 5.61% |
|  |  | 1651856 | rsA03_1651856 | E4 | 5.45 | 5.88% |
|  |  | 1656509 | rsA03_1656509 | E4 | 5.01 | 5.37% |
|  |  | 1656798 | rsA03_1656798 | E4 | 5.18 | 5.57% |
|  |  | 1657250 | rsA03_1657250 | E4 | 5.18 | 5.57% |
|  |  | 1657280 | rsA03_1657280 | E4 | 5.18 | 5.57% |
|  |  | 1657794 | rsA03_1657794 | E4 | 6.18 | 6.73% |
|  |  | 1658375 | rsA03_1658375 | E4 | 5.13 | 5.50% |
|  |  | 1659171 | rsA03_1659171 | E4 | 5.03 | 5.39% |
|  |  | 1671495 | rsA03_1671495 | E4 | 5.10 | 5.48% |
|  |  | 1672943 | rsA03_1672943 | E4 | 5.03 | 5.39% |
|  |  | 1672968 | rsA03_1672968 | E4 | 5.03 | 5.39% |
|  |  | 1711419 | rsA03_1711419 | E4 | 5.19 | 5.58% |
|  |  | 1777404 | rsA03_1777404 | E4 | 5.48 | 5.92% |
|  |  | 1793143 | rsA03_1793143 | E4 | 5.03 | 5.39% |
|  |  | 1795337 | rsA03_1795337 | E4 | 5.36 | 5.80% |
| qFBL-A04-1 | A04 | 5737292 | rsA04_5737292 | E1 | 5.01 | 5.38% |
| qFBL-A04-2 | A04 | 73095946 | rsA04_73095946 | E3 | 5.56 | 6.04% |
| qFBL-A05-1 | A05 | 30038530 | rsA05_30038530 | E6/BLUP | 5.61 | 6.06% |
| qFBL-A05-2 | A05 | 30476043 | rsA05_30476043 | E2 | 5.32 | 5.73% |
| qFBL-A05-3 | A05 | 36260755 | rsA05_36260755 | E3 | 5.14 | 5.56% |
| qFBL-A05-4 | A05 | 109209911 | rsA05_109209911 | E6/BLUP | 5.58 | 6.05% |
| qFBL-A06-1 | A06 | 62188100 | rsA06_62188100 | E5 | 5.29 | 5.68% |
| qFBL-A07-1 | A07 | 25235475 | rsA07_25235475 | E1 | 5.05 | 5.41% |
| qFBL-A07-2 | A07 | 46754152 | rsA07_46754152 | E2/E6/BLUP | 5.65 | 6.14% |
| qFBL-A07-3 | A07 | 90118145 | rsA07_90118145 | E2/E6/BLUP | 5.56 | 6.02% |
|  |  | 90118222 | rsA07_90118222 | E2/E6/BLUP | 5.45 | 5.90% |
| qFBL-A08-1 | A08 | 31866885 | rsA08_31866885 | E1 | 5.25 | 5.65% |
| qFBL-A08-2 | A08 | 36802561 | rsA08_36802561 | E3 | 5.43 | 5.87% |
| qFBL-A08-3 | A08 | 38046486 | rsA08_38046486 | E4 | 5.07 | 5.44% |
| qFBL-A08-4 | A08 | 38731888 | rsA08_38731888 | E3 | 5.10 | 5.51% |
| qFBL-A08-5 | A08 | 50421281 | rsA08_50421281 | E3 | 5.57 | 6.00% |
| qFBL-A08-6 | A08 | 53103303 | rsA08_53103303 | E1 | 5.53 | 5.97% |
| qFBL-A08-7 | A08 | 53462316 | rsA08_53462316 | BLUP | 5.22 | 5.60% |
| qFBL-A08-8 | A08 | 57963686 | rsA08_57963686 | E2 | 5.82 | 6.29% |
| qFBL-A08-9 | A08 | 69155335 | rsA08_69155335 | E5 | 5.06 | 5.44% |
| qFBL-A08-10 | A08 | 69581309 | rsA08_69581309 | E2 | 5.58 | 6.03% |
| qFBL-A08-11 | A08 | 72163761 | rsA08_72163761 | E3 | 5.25 | 5.62% |
| qFBL-A08-12 | A08 | 72657646 | rsA08_72657646 | E3 | 5.45 | 5.92% |
| qFBL-A08-13 | A08 | 88097554 | rsA08_88097554 | E4 | 5.28 | 5.66% |
| qFBL-A09-1 | A09 | 73048440 | rsA09_73048440 | E1 | 5.01 | 5.39% |
| qFBL-A10-1 | A10 | 5280095 | rsA10_5280095 | E2 | 5.14 | 5.50% |
| qFBL-A10-2 | A10 | 10116164 | rsA10_10116164 | E2/E5 | 5.51 | 5.96% |
| qFBL-A10-3 | A10 | 51997860 | rsA10_51997860 | E4 | 5.32 | 5.73% |
| qFBL-A10-4 | A10 | 77280367 | rsA10_77280367 | E4/E5/E6/BLUP | 6.02 | 6.53% |
|  |  | 77281000 | rsA10_77281000 | E5/E6 | 5.65 | 6.13% |
|  |  | 77285853 | rsA10_77285853 | E4/E5/E6/BLUP | 6.05 | 6.58% |
|  |  | 77286931 | rsA10_77286931 | E6/BLUP | 5.22 | 5.59% |
|  |  | 77287732 | rsA10_77287732 | E4/E5/E6/BLUP | 5.60 | 6.09% |
|  |  | 77297705 | rsA10_77297705 | E6/BLUP | 5.09 | 5.47% |
|  |  | 77298716 | rsA10_77298716 | E5/E6/BLUP | 5.49 | 5.93% |
|  |  | 77298868 | rsA10_77298868 | E5/E6/BLUP | 5.79 | 6.25% |
|  |  | 77299713 | rsA10_77299713 | E6/BLUP | 5.43 | 5.85% |
|  |  | 77299793 | rsA10_77299793 | E5/E6/BLUP | 5.56 | 6.00% |
|  |  | 77300288 | rsA10_77300288 | E4/E5/E6/BLUP | 7.03 | 7.75% |
|  |  | 77300485 | rsA10_77300485 | E6/BLUP | 5.09 | 5.42% |
|  |  | 77301107 | rsA10_77301107 | E5/E6/BLUP | 5.39 | 5.81% |
|  |  | 77305702 | rsA10_77305702 | E5/E6/BLUP | 5.51 | 5.97% |
|  |  | 77306649 | rsA10_77306649 | E6/BLUP | 5.23 | 5.65% |
|  |  | 77306677 | rsA10_77306677 | E6/BLUP | 5.23 | 5.65% |
|  |  | 77307152 | rsA10_77307152 | E2/E4/E5/E6/BLUP | 7.56 | 8.30% |
|  |  | 77307302 | rsA10_77307302 | E5/E6/BLUP | 6.32 | 6.89% |
|  |  | 77307637 | rsA10_77307637 | E5/E6/BLUP | 5.87 | 6.35% |
|  |  | 77308361 | rsA10_77308361 | E5/E6/BLUP | 6.51 | 7.12% |
|  |  | 77308747 | rsA10_77308747 | E5/E6/BLUP | 6.12 | 6.67% |
|  |  | 77385817 | rsA10_77385817 | E5/E6/BLUP | 5.68 | 6.16% |
|  |  | 77631335 | rsA10_77631335 | E2/E5/E6/BLUP | 5.97 | 6.49% |
|  |  | 77706709 | rsA10_77706709 | E5/E6/BLUP | 5.62 | 6.09% |
|  |  | 77729410 | rsA10_77729410 | E5/E6/BLUP | 5.92 | 6.49% |
|  |  | 77731950 | rsA10_77731950 | E6/BLUP | 5.28 | 5.67% |
| qFBL-A10-5 | A10 | 78399310 | rsA10_78399310 | E5/E6/BLUP | 5.40 | 5.81% |
|  |  | 78481486 | rsA10_78481486 | E6 | 5.06 | 5.44% |
| qFBL-A10-6 | A10 | 80676040 | rsA10_80676040 | E6/BLUP | 5.11 | 5.52% |
| qFBL-A10-7 | A10 | 81117896 | rsA10_81117896 | BLUP | 5.11 | 5.51% |
| qFBL-A10-8 | A10 | 84694773 | rsA10_84694773 | E5 | 5.10 | 5.46% |
|  |  | 84754890 | rsA10_84754890 | E5 | 5.10 | 5.46% |
| qFBL-A10-9 | A10 | 85661166 | rsA10_85661166 | E5 | 5.75 | 6.23% |
|  |  | 85713292 | rsA10_85713292 | E5 | 5.75 | 6.23% |
| qFBL-A10-10 | A10 | 88918850 | rsA10_88918850 | E5 | 5.41 | 5.84% |
| qFBL-A10-11 | A10 | 89282614 | rsA10_89282614 | E5 | 5.16 | 5.54% |
| qFBL-A11-1 | A11 | 70424727 | rsA11_70424727 | E1 | 5.45 | 5.89% |
| qFBL-A11-2 | A11 | 99585892 | rsA11_99585892 | E5 | 6.13 | 6.68% |
| qFBL-A12-1 | A12 | 90699572 | rsA12_90699572 | E5 | 7.08 | 7.77% |
| qFBL-A13-1 | A13 | 34388248 | rsA13_34388248 | E1 | 5.02 | 5.36% |
| qFBL-A13-2 | A13 | 102658949 | rsA13_102658949 | E3/E6/BLUP | 5.66 | 6.11% |
| qFBL-D03-1 | D03 | 3720768 | rsD03_3720768 | E4 | 6.25 | 6.81% |
| qFBL-D03-2 | D03 | 6430983 | rsD03_6430983 | E1 | 5.00 | 5.39% |
| qFBL-D03-3 | D03 | 6742345 | rsD03_6742345 | E5 | 5.56 | 6.02% |
| qFBL-D03-4 | D03 | 9273540 | rsD03_9273540 | E1/E4/E6/BLUP | 7.89 | 8.74% |
|  |  | 9277675 | rsD03_9277675 | E1 | 5.24 | 5.63% |
|  |  | 9363940 | rsD03_9363940 | E1 | 5.76 | 6.27% |
|  |  | 9456312 | rsD03_9456312 | E4/E6 | 5.11 | 5.47% |
|  |  | 9543296 | rsD03_9543296 | E1/E4 | 5.26 | 5.69% |
| qFBL-D03-5 | D03 | 9638928 | rsD03_9638928 | E1 | 5.26 | 5.63% |
|  |  | 9776383 | rsD03_9776383 | E1/E4 | 5.22 | 5.58% |
|  |  | 9777963 | rsD03_9777963 | E1 | 5.15 | 5.54% |
|  |  | 9790328 | rsD03_9790328 | E1 | 5.66 | 6.13% |
|  |  | 9872831 | rsD03_9872831 | E1/E4 | 5.39 | 5.84% |
|  |  | 9877695 | rsD03_9877695 | E1 | 5.06 | 5.47% |
| qFBL-D03-6 | D03 | 10041965 | rsD03_10041965 | E1 | 5.04 | 5.42% |
|  |  | 10118446 | rsD03_10118446 | E1 | 5.34 | 5.75% |
|  |  | 10119695 | rsD03_10119695 | E1 | 5.34 | 5.75% |
| qFBL-D03-7 | D03 | 24579450 | rsD03_24579450 | E2 | 5.70 | 6.20% |
| qFBL-D03-8 | D03 | 27869854 | rsD03_27869854 | BLUP | 5.04 | 5.39% |
| qFBL-D03-9 | D03 | 30082291 | rsD03_30082291 | E4/E6 | 5.05 | 5.40% |
| qFBL-D03-10 | D03 | 31213926 | rsD03_31213926 | E1 | 5.08 | 5.45% |
|  |  | 31281011 | rsD03_31281011 | E1 | 5.01 | 5.40% |
|  |  | 31377854 | rsD03_31377854 | E1 | 5.17 | 5.57% |
|  |  | 31377884 | rsD03_31377884 | E1 | 5.14 | 5.54% |
|  |  | 31445511 | rsD03_31445511 | E1 | 5.04 | 5.42% |
|  |  | 31446061 | rsD03_31446061 | E1 | 5.03 | 5.40% |
| qFBL-D03-11 | D03 | 31524007 | rsD03_31524007 | E1 | 5.26 | 5.65% |
|  |  | 31542702 | rsD03_31542702 | E1 | 5.01 | 5.37% |
| qFBL-D03-12 | D03 | 31886930 | rsD03_31886930 | E1 | 5.12 | 5.52% |
|  |  | 31955065 | rsD03_31955065 | E1 | 5.81 | 6.32% |
| qFBL-D03-13 | D03 | 32415522 | rsD03_32415522 | E1 | 5.60 | 6.06% |
|  |  | 32494581 | rsD03_32494581 | E1 | 5.37 | 5.79% |
| qFBL-D03-14 | D03 | 33921827 | rsD03_33921827 | E1 | 5.70 | 6.17% |
|  |  | 34014232 | rsD03_34014232 | E1 | 5.84 | 6.31% |
| qFBL-D03-15 | D03 | 36696073 | rsD03_36696073 | E1 | 6.14 | 6.67% |
| qFBL-D03-16 | D03 | 37595985 | rsD03_37595985 | E1/E4 | 8.10 | 9.01% |
| qFBL-D03-17 | D03 | 37913409 | rsD03_37913409 | E6 | 5.28 | 5.69% |
|  |  | 37922251 | rsD03_37922251 | E1/E2/E4/E6/BLUP | 9.76 | 10.86% |
|  |  | 37950264 | rsD03_37950264 | E1/E4/E6 | 8.90 | 9.88% |
|  |  | 37966392 | rsD03_37966392 | E1/E2/E4/E6/BLUP | 8.79 | 9.72% |
|  |  | 38010395 | rsD03_38010395 | E1/E4 | 9.20 | 10.23% |
|  |  | 38030754 | rsD03_38030754 | E1/E2/E4/E6/BLUP | 9.11 | 10.12% |
|  |  | 38060837 | rsD03_38060837 | E1/E2/E4/E6/BLUP | 9.69 | 10.75% |
|  |  | 38110251 | rsD03_38110251 | E1/E4/E6/BLUP | 8.67 | 9.66% |
|  |  | 38124641 | rsD03_38124641 | E1/E4 | 7.37 | 8.10% |
|  |  | 38189392 | rsD03_38189392 | E1/E2/E4/E6/BLUP | 9.88 | 11.02% |
|  |  | 38197905 | rsD03_38197905 | E1 | 6.87 | 7.55% |
|  |  | 38205704 | rsD03_38205704 | E1/E4/E6/BLUP | 9.32 | 10.40% |
|  |  | 38219036 | rsD03_38219036 | E1/E2/E4/E6/BLUP | 8.94 | 9.91% |
|  |  | 38257551 | rsD03_38257551 | E1 | 7.25 | 8.01% |
|  |  | 38317839 | rsD03_38317839 | E1 | 7.97 | 8.83% |
|  |  | 38375661 | rsD03_38375661 | E1/E4/E6 | 8.98 | 9.97% |
|  |  | 38384624 | rsD03_38384624 | E1/E2/E4/E6/BLUP | 9.53 | 10.64% |
|  |  | 38445420 | rsD03_38445420 | E1/E2/E4/E6/BLUP | 9.57 | 10.64% |
|  |  | 38451719 | rsD03_38451719 | E1/E4/E6 | 9.15 | 10.19% |
|  |  | 38470193 | rsD03_38470193 | E1/E4/E6 | 9.01 | 10.02% |
|  |  | 38474154 | rsD03_38474154 | E1/E2/E4/E6/BLUP | 9.49 | 10.54% |
|  |  | 38491965 | rsD03_38491965 | E1/E4/E6/BLUP | 9.43 | 10.53% |
|  |  | 38533246 | rsD03_38533246 | E1/E2/E4 | 8.60 | 9.52% |
|  |  | 38550779 | rsD03_38550779 | E1 | 5.74 | 6.18% |
|  |  | 38571296 | rsD03_38571296 | E1/E4/E6/BLUP | 10.76 | 11.96% |
|  |  | 38598523 | rsD03_38598523 | E1 | 6.32 | 6.89% |
|  |  | 38685508 | rsD03_38685508 | E1/E4/E6/BLUP | 7.78 | 8.56% |
|  |  | 38746305 | rsD03_38746305 | E1/E4/E6/BLUP | 9.57 | 10.61% |
|  |  | 38782780 | rsD03_38782780 | E1/E2/E4/E6/BLUP | 8.97 | 9.94% |
|  |  | 38801793 | rsD03_38801793 | E1/E4/E6/BLUP | 8.51 | 9.40% |
|  |  | 38843446 | rsD03_38843446 | E1/E4 | 8.15 | 9.01% |
|  |  | 38950556 | rsD03_38950556 | E1/E4/E6/BLUP | 8.48 | 9.45% |
|  |  | 38960617 | rsD03_38960617 | E1/E2/E4/E6/BLUP | 9.33 | 10.33% |
|  |  | 38965401 | rsD03_38965401 | E1 | 6.66 | 7.29% |
|  |  | 38966194 | rsD03_38966194 | E1/E4/E6/BLUP | 8.82 | 9.78% |
|  |  | 39076765 | rsD03_39076765 | E1/E2/E4 | 7.61 | 8.42% |
|  |  | 39085137 | rsD03_39085137 | E1 | 7.88 | 8.75% |
|  |  | 39137197 | rsD03_39137197 | E1/E4/E6 | 8.95 | 9.93% |
|  |  | 39170262 | rsD03_39170262 | E1/E2/E4/E6/BLUP | 9.76 | 10.86% |
|  |  | 39273424 | rsD03_39273424 | E1 | 6.63 | 7.25% |
|  |  | 39307341 | rsD03_39307341 | E1 | 7.08 | 7.80% |
| qFBL-D04-1 | D04 | 7779899 | rsD04_7779899 | E3 | 5.03 | 5.38% |
| qFBL-D04-2 | D04 | 22051324 | rsD04_22051324 | E5 | 5.49 | 5.93% |
| qFBL-D07-1 | D07 | 6605218 | rsD07_6605218 | E1 | 5.52 | 5.94% |
|  |  | 6608289 | rsD07_6608289 | E1 | 5.06 | 5.46% |
|  |  | 6612506 | rsD07_6612506 | E1 | 5.47 | 5.94% |
|  |  | 6628051 | rsD07_6628051 | E1 | 5.89 | 6.44% |
|  |  | 6655224 | rsD07_6655224 | E1 | 5.07 | 5.43% |
|  |  | 6656751 | rsD07_6656751 | E1 | 5.71 | 6.17% |
|  |  | 6659269 | rsD07_6659269 | E1 | 5.74 | 6.22% |
| qFBL-D07-2 | D07 | 7105100 | rsD07_7105100 | E6/BLUP | 5.44 | 5.88% |
|  |  | 7105108 | rsD07_7105108 | BLUP | 5.28 | 5.67% |
| qFBL-D07-3 | D07 | 57581338 | rsD07_57581338 | E3 | 5.66 | 6.12% |
| qFBL-D08-1 | D08 | 16799130 | rsD08_16799130 | E2 | 5.17 | 5.54% |
| qFBL-D09-1 | D09 | 43935233 | rsD09_43935233 | E3 | 5.87 | 6.39% |
| qFBL-D10-1 | D10 | 14268841 | rsD10_14268841 | E1 | 5.21 | 5.59% |
| qFBL-D10-2 | D10 | 15375753 | rsD10_15375753 | E1 | 5.02 | 5.35% |
| qFBL-D10-3 | D10 | 58005633 | rsD10_58005633 | E5 | 5.43 | 5.83% |
|  |  | 58247192 | rsD10_58247192 | E1/E4/E5/E6/BLUP | 6.61 | 7.23% |
|  |  | 58265573 | rsD10_58265573 | E1/E4/E6/BLUP | 5.96 | 6.47% |
|  |  | 58267281 | rsD10_58267281 | E1/E5/E6/BLUP | 5.87 | 6.38% |
| qFBL-D10-4 | D10 | 58492441 | rsD10_58492441 | E1/E6/BLUP | 5.49 | 5.93% |
|  |  | 58608098 | rsD10_58608098 | E1 | 5.02 | 5.40% |
|  |  | 58771724 | rsD10_58771724 | E5 | 5.13 | 5.50% |
|  |  | 58776113 | rsD10_58776113 | E5 | 5.01 | 5.37% |
| qFBL-D11-1 | D11 | 4050257 | rsD11_4050257 | E6 | 5.04 | 5.37% |
| qFBL-D11-2 | D11 | 70846056 | rsD11_70846056 | E5 | 5.01 | 5.36% |
|  |  | 70846586 | rsD11_70846586 | E5 | 5.04 | 5.39% |
|  |  | 70846624 | rsD11_70846624 | E5 | 5.05 | 5.42% |
|  |  | 70846695 | rsD11_70846695 | E5 | 5.22 | 5.62% |
|  |  | 70846772 | rsD11_70846772 | E5 | 5.34 | 5.74% |
|  |  | 70846778 | rsD11_70846778 | E5 | 5.34 | 5.74% |
|  |  | 70846782 | rsD11_70846782 | E5 | 5.05 | 5.39% |
|  |  | 70846797 | rsD11_70846797 | E5 | 5.05 | 5.39% |
|  |  | 70846930 | rsD11_70846930 | E5 | 5.13 | 5.50% |
|  |  | 70846995 | rsD11_70846995 | E5 | 5.04 | 5.40% |
|  |  | 70847062 | rsD11_70847062 | E5 | 5.08 | 5.44% |
| qFBL-D11-3 | D11 | 61197451 | rsD12_61197451 | E6/BLUP | 5.31 | 5.77% |
|  |  | 61197836 | rsD12_61197836 | E6/BLUP | 5.62 | 6.12% |
|  |  | 61198126 | rsD12_61198126 | E1/E6/BLUP | 5.63 | 6.13% |
|  |  | 61198209 | rsD12_61198209 | E6/BLUP | 5.88 | 6.40% |
|  |  | 61198854 | rsD12_61198854 | E6/BLUP | 5.54 | 6.02% |
|  |  | 61203626 | rsD12_61203626 | BLUP | 5.01 | 5.35% |
|  |  | 61204609 | rsD12_61204609 | BLUP | 5.01 | 5.33% |
|  |  | 61214344 | rsD12_61214344 | E6/BLUP | 5.69 | 6.12% |
|  |  | 61214495 | rsD12_61214495 | E6/BLUP | 5.29 | 5.72% |
|  |  | 61242721 | rsD12_61242721 | BLUP | 5.00 | 5.39% |

| **Table S6: Summary of the annotations of 10 genes on qFBL-A10-4.** | | |
| --- | --- | --- |
| *G.hirsutum gene ID* | Arabidopsis ID | ArabDesc |
| Ghir_A10G014300 | AT5G67550 | unknown protein |
| Ghir_A10G014310 | AT3G07565 | Protein of unknown function (DUF3755) |
| Ghir_A10G014320 | AT2G43490 | Ypt/Rab-GAP domain of gyp1p superfamily protein |
| Ghir_A10G014330 | AT3G59580 | Plant regulator RWP-RK family protein |
| Ghir_A10G014340 | AT2G30710 | Ypt/Rab-GAP domain of gyp1p superfamily protein |
| Ghir_A10G014350 | AT1G03940 | HXXXD-type acyl-transferase family protein |
| Ghir_A10G014360 | AT1G09780 | Phosphoglycerate mutase, 2,3-bisphosphoglycerate-independent |
| Ghir_A10G014370 | AT1G74190 | receptor like protein 15 |
| Ghir_A10G014380 | AT2G25470 | receptor like protein 21 |
| Ghir_A10G014390 | AT1G74190 | receptor like protein 15 |

| **Table S7: Summary of the annotations of 49 genes on qFBL-D03-17.** | | |
| --- | --- | --- |
| *G.hirsutum gene ID* | Arabidopsis ID | ArabDesc |
| Ghir_D03G010990 | NA | NA |
| Ghir_D03G011000 | AT2G24762 | glutamine dumper 4 |
| Ghir_D03G011010 | AT3G02380 | CONSTANS-like 2 |
| Ghir_D03G011020 | AT5G08160 | serine/threonine protein kinase 3 |
| Ghir_D03G011030 | AT5G08180 | Ribosomal protein L7Ae/L30e/S12e/Gadd45 family protein |
| Ghir_D03G011040 | AT5G47920 | unknown protein |
| Ghir_D03G011050 | AT1G74030 | enolase 1 |
| Ghir_D03G011060 | AT5G23040 | Protein of unknown function (DUF3353) |
| Ghir_D03G011070 | AT5G23050 | acyl-activating enzyme 17 |
| Ghir_D03G011080 | AT3G12300 | unknown protein |
| Ghir_D03G011090 | NA | NA |
| Ghir_D03G011100 | NA | NA |
| Ghir_D03G011110 | AT1G74055 | unknown protein |
| Ghir_D03G011120 | AT1G68710 | ATPase E1-E2 type family protein |
| Ghir_D03G011130 | AT5G23080 | TGH \| SWAP (Suppressor-of-White-APricot)/surp domain-containing protein |
| Ghir_D03G011140 | AT1G18550 | ATP binding microtubule motor family protein |
| Ghir_D03G011150 | AT5G64370 | BETA-UP, PYD3 \| beta-ureidopropionase |
| Ghir_D03G011160 | AT4G23160 | cysteine-rich RLK (RECEPTOR-like protein kinase) 8 |
| Ghir_D03G011170 | AT3G18600 | P-loop containing nucleoside triphosphate hydrolases superfamily protein |
| Ghir_D03G011180 | AT4G12690 | Plant protein of unknown function (DUF868) |
| Ghir_D03G011190 | AT5G51520 | Plant invertase/pectin methylesterase inhibitor superfamily protein |
| Ghir_D03G011200 | NA | NA |
| Ghir_D03G011210 | AT4G24730 | Calcineurin-like metallo-phosphoesterase superfamily protein |
| Ghir_D03G011220 | AT3G48310 | cytochrome P450 |
| Ghir_D03G011230 | AT4G24740 | FUS3-complementing gene 2 |
| Ghir_D03G011240 | AT3G26310 | cytochrome P450 |
| Ghir_D03G011250 | AT3G57520 | seed imbibition 2 |
| Ghir_D03G011260 | AT4G24770 | 31-kDa RNA binding protein |
| Ghir_D03G011270 | AT3G48330 | protein-l-isoaspartate methyltransferase 1 |
| Ghir_D03G011280 | AT3G01680 | unknown protein |
| Ghir_D03G011290 | AT2G31880 | Leucine-rich repeat protein kinase family protein |
| Ghir_D03G011300 | AT1G24530 | Transducin/WD40 repeat-like superfamily protein |
| Ghir_D03G011310 | AT5G50260 | Cysteine proteinases superfamily protein |
| Ghir_D03G011320 | AT1G67750 | Symbols: \| Pectate lyase family protein |
| Ghir_D03G011330 | AT5G02850 | hydroxyproline-rich glycoprotein family protein |
| Ghir_D03G011340 | AT4G13940 | S-adenosyl-L-homocysteine hydrolase |
| Ghir_D03G011350 | AT3G48380 | Peptidase C78 |
| Ghir_D03G011360 | AT1G06990 | GDSL-like Lipase/Acylhydrolase superfamily protein |
| Ghir_D03G011370 | AT2G01050 | zinc ion binding;nucleic acid binding |
| Ghir_D03G011380 | AT5G63190 | MA3 domain-containing protein |
| Ghir_D03G011390 | AT3G48430 | relative of early flowering 6 |
| Ghir_D03G011400 | AT2G36850 | glucan synthase-like 8 |
| Ghir_D03G011410 | AT1G13195 | RING/U-box superfamily protein |
| Ghir_D03G011420 | AT2G24960 | unknown protein |
| Ghir_D03G011430 | AT5G50300 | Xanthine/uracil permease family protein |
| Ghir_D03G011440 | AT4G24820 | 26S proteasome, regulatory subunit Rpn7 |
| Ghir_D03G011450 | AT5G63320 | NPX1 \| nuclear protein X1 |
| Ghir_D03G011460 | AT1G15910 | XH/XS domain-containing protein |
| Ghir_D03G011470 | AT5G50360 | unknown protein |

| **Table S8: The aggregation of excellent haplotype number of two candidate genes in the natural populations.** | | | | | |
| --- | --- | --- | --- | --- | --- |
| Line | Cultivar/Germplasm line | STAGE | Ghir_A10G014390 | Ghir_D03G011390 | Number of Elite Alleles |
| S2 | Zhong40418 | S4 | TT | AA | 0 |
| S3 | Zhong40618 | S4 | TT | AA | 0 |
| S4 | Zhong40612 | S4 | TT | NA | 0 |
| S5 | Zhong51811 | S4 | TT | AA | 0 |
| S6 | CRI94A1822 | S4 | CC | GG | 2 |
| S7 | Zhong61832 | S4 | CC | GG | 2 |
| S8 | Zhong71239 | S4 | CC | GG | 2 |
| S9 | PB12-1-10 | S4 | CC | GG | 2 |
| S10 | PB12-1-7 | S4 | CC | GG | 2 |
| S11 | PB12-1-8 | S4 | CC | GG | 2 |
| S12 | Zhong1476 | S4 | CC | GG | 2 |
| S13 | Zhong151222 | S4 | TT | AA | 0 |
| S14 | Zhong152201 | S4 | TT | GG | 1 |
| S15 | Zhong152201SQ | S4 | NA | GG | 1 |
| S16 | Zhong152214 | S4 | CC | GG | 2 |
| S17 | Zhong152224 | S4 | CC | GG | 2 |
| S18 | SS2011 | S4 | CC | GG | 2 |
| S19 | CQ2012-3 | S4 | CC | GG | 2 |
| S20 | CQ2012-4 | S4 | CC | GG | 2 |
| S21 | Zhong29T41 | NA | CC | GG | 2 |
| S22 | Zhong29T42 | NA | CC | GG | 2 |
| S23 | L426 | NA | CC | GG | 2 |
| S24 | K640 | S4 | CC | GG | 2 |
| S25 | N82 | NA | CC | GG | 2 |
| S28 | sGK16 | S4 | TT | GG | 1 |
| S30 | Baimian 17 | S4 | CC | GG | 2 |
| S36 | Han656 | S4 | CC | GG | 2 |
| S37 | Han559 | S4 | CC | GG | 2 |
| S38 | Han667 | NA | CC | GG | 2 |
| S39 | Han686 | S4 | CC | GG | 2 |
| S40 | Han9609 | NA | TT | GG | 1 |
| S49 | Liaomian5 | S2 | TT | GG | 1 |
| S50 | Liaomian6 | S2 | CC | GG | 2 |
| S51 | Liaomian7 | S2 | CC | GG | 2 |
| S55 | Lumianyan19 | S4 | CC | NA | 1 |
| S56 | Lumian2153 | S4 | CC | GG | 2 |
| S61 | Shizao3 | S4 | CC | GG | 2 |
| S62 | X13-7 | S4 | CC | GG | 2 |
| S64 | Xiazao1 | S3 | CC | GG | 2 |
| S65 | Xiazao2 | S4 | CC | GG | 2 |
| S67 | Xinluzao11 | S3 | CC | GG | 2 |
| S68 | Xinluzao36 | S4 | TT | AA | 0 |
| S69 | Xinluzao3 | S3 | CC | GG | 2 |
| S80 | Yuzao9110 | S4 | CC | GG | 2 |
| S81 | Jinmian57 | S4 | CC | GG | 2 |
| S83 | Zhong416 | NA | CC | AA | 1 |
| S86 | Zhong213 | S4 | CC | GG | 2 |
| S88 | CRI10 | S2 | CC | GG | 2 |
| S89 | CRI14 | S3 | CC | AA | 1 |
| S92 | CRI24 | S3 | TT | GG | 1 |
| S93 | CRI27 | S3 | CC | GG | 2 |
| S94 | CRI30 | S3 | TT | GG | 1 |
| S95 | CRI36 | S3 | CC | GG | 2 |
| S97 | CRI37 | S3 | CC | GG | 2 |
| S98 | CRI42 | S4 | TT | GG | 1 |
| S99 | CRI50 | S4 | CC | GG | 2 |
| S102 | CRI74 | S4 | CC | GG | 2 |
| S187 | XianⅢ9704 | NA | CC | GG | 2 |
| S190 | Bazhou5409 | NA | CC | GG | 2 |
| S191 | Yumian1 | S4 | CC | GG | 2 |
| S224 | Ken27-3 | NA | TT | AA | 0 |
| S229 | Xinluzao28 | S4 | CC | GG | 2 |
| S243 | Xinluzao48 | S4 | CC | GG | 2 |
| S267 | Xinluzhong27 | S4 | CC | GG | 2 |
| S317 | Gangmian1 | S2 | CC | GG | 2 |
| S321 | Yishuhong | S2 | TT | GG | 1 |
| S334 | Dunmian2 | S3 | TT | AA | 0 |
| S335 | Ganmian2 | S2 | TT | AA | 0 |
| S1 | Zhong2191 | S4 | CC | GG | 2 |
| S26 | P21-6-7 | NA | CC | GG | 2 |
| S27 | Shannong SF06 | S4 | CC | GG | 2 |
| S29 | V321-20-14 | NA | CC | GG | 2 |
| S31 | Chaoaoyang 1 | S1 | CC | GG | 2 |
| S32 | Deltapine 20 | S3 | CC | GG | 2 |
| S33 | De 97-047 | NA | CC | GG | 2 |
| S34 | Guannong 1 | S1 | CC | GG | 2 |
| S35 | Han2490 | S4 | TT | AA | 0 |
| S41 | Heishanmian1 | S2 | TT | GG | 1 |
| S42 | Jinmian3 | S3 | TT | GG | 1 |
| S43 | Jinmian10 | S3 | CC | GG | 2 |
| S44 | Jinmian21 | S3 | CC | GG | 2 |
| S45 | Jinmian23 | S3 | CC | GG | 2 |
| S46 | Jinmian5 | S2 | TT | AA | 0 |
| S47 | Liaomian10 | S3 | CC | GG | 2 |
| S48 | Liaomian17 | S4 | TT | GG | 1 |
| S52 | Liaomian9 | S2 | TT | GG | 1 |
| S53 | Liaoyangduanjie | S1 | CC | GG | 2 |
| S54 | Lu154 | S4 | CC | GG | 2 |
| S57 | Nongken5 | S2 | TT | AA | 0 |
| S58 | Shan70 | NA | CC | GG | 2 |
| S59 | Shizao1 | S4 | CC | GG | 2 |
| S60 | Shizao2 | S4 | CC | GG | 2 |
| S63 | X25TF | S4 | CC | GG | 2 |
| S66 | Xiazao3 | S4 | CC | GG | 2 |
| S70 | Xinluzao42 | S4 | CC | GG | 2 |
| S71 | Xinluzao45 | S4 | CC | GG | 2 |
| S72 | Xinluzao4 | S3 | CC | GG | 2 |
| S73 | Xinluzao6 | S3 | CC | GG | 2 |
| S74 | Xinluzao8 | S3 | CC | GG | 2 |
| S75 | Xinluzao9 | S3 | CC | GG | 2 |
| S76 | Mianxiang368 | S4 | CC | GG | 2 |
| S77 | Yu1335 | S4 | CC | GG | 2 |
| S78 | Yumian12 | S3 | CC | GG | 2 |
| S79 | Yuzao8E13 | S3 | CC | AA | 1 |
| S82 | YunzaoN95 | S4 | CC | AA | 1 |
| S84 | Zhong425-5 | NA | CC | GG | 2 |
| S85 | Zhong716 | S3 | CC | GG | 2 |
| S87 | Zhongchuang 88 | S4 | CC | GG | 2 |
| S90 | CRI16 | S3 | TT | GG | 1 |
| S91 | CRI20 | S3 | CC | GG | 2 |
| S96 | Han256 | S4 | CC | GG | 2 |
| S100 | CRI58 | S4 | TT | GG | 1 |
| S101 | CRI64 | S4 | TT | AA | 0 |
| S103 | Zhong00776 | S4 | CC | AA | 1 |
| S104 | Zhong20398 | S4 | CC | GG | 2 |
| S105 | Zhong61930 | S4 | CC | AA | 1 |
| S106 | Zhong61995 | S4 | CC | AA | 1 |
| S107 | Zhong102909 | S4 | TT | GG | 1 |
| S108 | Zhong103026 | S4 | CC | GG | 2 |
| S109 | Zhong103028 | S4 | CC | GG | 2 |
| S110 | Zhong103030 | S4 | CC | GG | 2 |
| S111 | Zhong103032 | S4 | TT | GG | 1 |
| S112 | Zhong103075 | S4 | TT | AA | 0 |
| S114 | Zhong103425 | S4 | CC | GG | 2 |
| S115 | Zhong103297 | S4 | CC | GG | 2 |
| S116 | Zhong109056 | S4 | CC | GG | 2 |
| S117 | CPB12-1-7 | S4 | CC | GG | 2 |
| S118 | CPB12-1-9 | S4 | CC | GG | 2 |
| S119 | CPB12-2-7 | S4 | CC | GG | 2 |
| S120 | 298 | NA | CC | GG | 2 |
| S121 | Zhong602186 | S4 | CC | AA | 1 |
| S122 | CG3020-1 | NA | CC | GG | 2 |
| S123 | CG3020-3 | NA | TT | GG | 1 |
| S124 | FM1735 | NA | TT | AA | 0 |
| S126 | Zhongzhimian2 | S4 | TT | GG | 1 |
| S127 | H109 | S3 | CC | GG | 2 |
| S129 | LIH33 | NA | TT | GG | 1 |
| S130 | Phy-7 | NA | CC | AA | 1 |
| S131 | STS458 | NA | CC | AA | 1 |
| S132 | TM-1 | NA | CC | GG | 2 |
| S133 | Aoshimian 6 | S4 | CC | GG | 2 |
| S134 | Baimian985 | S4 | TT | GG | 1 |
| S135 | Cang198 | S4 | TT | GG | 1 |
| S136 | CBB | S4 | CC | GG | 2 |
| S137 | CNB | S4 | CC | AA | 1 |
| S138 | Deltapine14 | S1 | CC | AA | 1 |
| S139 | Deltapine15 | S1 | CC | GG | 2 |
| S140 | Fanmian3 | S4 | TT | GG | 1 |
| S141 | Ganzao109 | NA | CC | AA | 1 |
| S142 | Guoxinmian11 | S4 | CC | GG | 2 |
| S143 | Han7860 | S4 | CC | AA | 1 |
| S144 | Ji4025 | S4 | CC | GG | 2 |
| S145 | Jimian 26 | S3 | CC | AA | 1 |
| S146 | BM03 | NA | CC | AA | 1 |
| S147 | Kelin098 | S4 | CC | AA | 1 |
| S148 | Liaomian23 | S4 | CC | AA | 1 |
| S149 | Liaomian27 | S4 | CC | GG | 2 |
| S150 | Liaomian28 | S4 | CC | AA | 1 |
| S151 | Lu05R59 | S4 | TT | GG | 1 |
| S152 | Lu7619 | S4 | CC | GG | 2 |
| S153 | Lumianyan17 | S4 | CC | GG | 2 |
| S154 | Lumianyan21 | S4 | CC | GG | 2 |
| S155 | Lumianyan28 | S4 | CC | GG | 2 |
| S156 | Lumianyan36 | S4 | CC | GG | 2 |
| S157 | Lumianyan38 | S4 | TT | AA | 0 |
| S158 | Miaobao21 | S4 | CC | GG | 2 |
| S159 | Renhe39 | S4 | NA | GG | 1 |
| S160 | Rihuimian6 | S4 | CC | GG | 2 |
| S165 | Xinzhimian5 | S4 | TT | GG | 1 |
| S166 | Yinhuashu | S4 | TT | GG | 1 |
| S167 | You009 | NA | CC | GG | 2 |
| S168 | Zhong109 | S4 | CC | GG | 2 |
| S169 | CRI17 | S3 | CC | GG | 2 |
| S170 | CRI19 | S3 | TT | GG | 1 |
| S171 | CRI43 | S4 | CC | GG | 2 |
| S172 | CRI60 | S4 | TT | GG | 1 |
| S173 | Zhong662 | S3 | CC | GG | 2 |
| S174 | Zhong679 | S4 | CC | GG | 2 |
| S175 | Zhong69 | S4 | CC | GG | 2 |
| S177 | CRI 94A915 | S4 | CC | GG | 2 |
| S178 | CRI12 | S3 | CC | GG | 2 |
| S179 | CRI35 | S3 | CC | GG | 2 |
| S180 | CRI41 | S4 | CC | GG | 2 |
| S181 | CRI45 | S4 | CC | AA | 1 |
| S182 | CRI49 | S4 | CC | GG | 2 |
| S183 | CRI7 | S2 | CC | GG | 2 |
| S184 | Zhongzhimian 8 | S2 | CC | GG | 2 |
| S185 | Zhongzhimian GD89 | S4 | CC | GG | 2 |
| S186 | Shan920346 | NA | CC | GG | 2 |
| S188 | US-1 | NA | CC | GG | 2 |
| S189 | Kyrgyzstan cotton | NA | CC | GG | 2 |
| S192 | Huazhong910102 | NA | CC | GG | 2 |
| S193 | Israel cotton | NA | CC | GG | 2 |
| S194 | Ken6614 | NA | TT | GG | 1 |
| S195 | Ken0074 | NA | NA | GG | 1 |
| S196 | Chuan239-1 | NA | CC | GG | 2 |
| S197 | Bamian3 | S4 | CC | GG | 2 |
| S198 | Chuan338 | NA | CC | GG | 2 |
| S199 | Chuan267 | NA | CC | GG | 2 |
| S201 | Chuanjian1 | S2 | TT | GG | 1 |
| S202 | Xinluzao 2 | S3 | CC | GG | 2 |
| S203 | Xinluzao10 | S3 | CC | GG | 2 |
| S205 | Xinluzao13 | S4 | CC | GG | 2 |
| S206 | Xinluzao15 | S4 | CC | GG | 2 |
| S207 | Xinluzao16 | S4 | CC | GG | 2 |
| S208 | Xinluzao17 | S4 | CC | GG | 2 |
| S209 | Xinluzao18 | S4 | TT | AA | 0 |
| S210 | Xinluzao19 | S4 | TT | GG | 1 |
| S211 | Xinluzao20 | S4 | TT | GG | 1 |
| S212 | Xinluzao21 | S4 | TT | GG | 1 |
| S213 | Xinluzao22 | S4 | TT | AA | 0 |
| S215 | Xinluzao24 | S4 | CC | AA | 1 |
| S216 | Yumian5 | S3 | CC | GG | 2 |
| S217 | Yumian18 | S3 | CC | GG | 2 |
| S218 | Yumian21 | S3 | CC | AA | 1 |
| S219 | Yun1729 | S3 | TT | GG | 1 |
| S220 | Zhemian11 | S3 | CC | GG | 2 |
| S221 | Tkuo | NA | CC | GG | 2 |
| S222 | Bo425 | NA | TT | GG | 1 |
| S223 | Xinluzhong 60 | S4 | TT | AA | 0 |
| S225 | B-3 | NA | CC | GG | 2 |
| S226 | Xinluzao25 | S4 | CC | AA | 1 |
| S227 | Xinluzao26 | S4 | TT | GG | 1 |
| S228 | Xinluzao27 | S4 | TT | GG | 1 |
| S230 | Xinluzao29 | S4 | CC | GG | 2 |
| S231 | Xinluzao30 | S4 | CC | GG | 2 |
| S232 | Xinluzao32 | S4 | TT | GG | 1 |
| S233 | Xinluzao33 | S4 | TT | GG | 1 |
| S234 | Xinluzao34 | S4 | CC | GG | 2 |
| S235 | Xinluzao35 | S4 | CC | GG | 2 |
| S236 | Xinluzao37 | S4 | CC | GG | 2 |
| S237 | Xinluzao38 | S4 | CC | GG | 2 |
| S238 | Xinluzao39 | S4 | CC | GG | 2 |
| S239 | Xinluzao40 | S4 | CC | GG | 2 |
| S240 | Xinluzao41 | S4 | CC | GG | 2 |
| S241 | Xinluzao46 | S4 | CC | GG | 2 |
| S242 | Xinluzao47 | S4 | CC | GG | 2 |
| S245 | Xinluzao50 | S4 | CC | GG | 2 |
| S246 | Xinluzao51 | S4 | CC | AA | 1 |
| S247 | Xinluzhong1 | S3 | CC | GG | 2 |
| S248 | Xinluzhong3 | S3 | CC | GG | 2 |
| S249 | Xinluzhong4 | S3 | CC | GG | 2 |
| S250 | Xinluzhong5 | S3 | CC | GG | 2 |
| S251 | Xinluzhong6 | S3 | CC | GG | 2 |
| S252 | Xinluzhong7 | S3 | CC | GG | 2 |
| S253 | Xinluzhong8 | S3 | CC | GG | 2 |
| S254 | Xinluzhong9 | S4 | TT | GG | 1 |
| S255 | Xinluzhong10 | S4 | CC | GG | 2 |
| S256 | Xinluzhong12 | S4 | TT | AA | 0 |
| S257 | Xinluzhong13 | S4 | CC | AA | 1 |
| S258 | Xinluzhong14 | S4 | CC | GG | 2 |
| S259 | Xinluzhong15 | S4 | TT | AA | 0 |
| S261 | Xinluzhong17 | S4 | CC | GG | 2 |
| S263 | Xinluzhong20 | S4 | TT | AA | 0 |
| S264 | Xinluzhong21 | S4 | CC | GG | 2 |
| S265 | Xinluzhong22 | S4 | CC | GG | 2 |
| S266 | Xinluzhong26 | S4 | TT | GG | 1 |
| S268 | Xinluzhong28 | S4 | CC | GG | 2 |
| S269 | Xinluzhong30 | S4 | CC | GG | 2 |
| S270 | Xinluzhong32 | S4 | CC | AA | 1 |
| S271 | Xinluzhong34 | S4 | CC | GG | 2 |
| S272 | Xinluzhong35 | S4 | CC | GG | 2 |
| S273 | Xinluzhong40 | S4 | CC | GG | 2 |
| S275 | Xinluzhong45 | S4 | CC | GG | 2 |
| S276 | Xinluzhong46 | S4 | CC | GG | 2 |
| S278 | Kangcaoganlin | S3 | CC | GG | 2 |
| S279 | Jiangyin1 | NA | CC | GG | 2 |
| S281 | Jinken1042 | S4 | CC | GG | 2 |
| S283 | Junmian1 | S2 | CC | GG | 2 |
| S284 | Huiyuan717 | S4 | CC | GG | 2 |
| S285 | Yunzao219 | NA | CC | GG | 2 |
| S286 | Yunzao33-356 | NA | TT | AA | 0 |
| S287 | Jinmian2 | S2 | TT | AA | 0 |
| S288 | Chaoyangmian2 | S2 | CC | AA | 1 |
| S289 | Dunhuang77-116 | NA | TT | AA | 0 |
| S290 | Ganmian4 | S3 | TT | AA | 0 |
| S291 | Guannongzao C-50 | NA | TT | AA | 0 |
| S292 | Guannongchangzao B14 | NA | TT | AA | 0 |
| S293 | Yanzao1 | NA | CC | GG | 2 |
| S294 | Yanzao2 | NA | TT | GG | 1 |
| S295 | Jinmian6 | S3 | TT | GG | 1 |
| S296 | Jinken69-2 | S4 | TT | GG | 1 |
| S297 | Jinken148-39 | S4 | TT | GG | 1 |
| S298 | Zhuangjiahan102 | NA | CC | AA | 1 |
| S299 | Yinshan4 | S4 | CC | AA | 1 |
| S300 | Ejing1 | S2 | TT | AA | 0 |
| S301 | Simian3 | S3 | TT | AA | 0 |
| S302 | Stoneville 4B | S1 | TT | AA | 0 |
| S303 | Jimian10 | S3 | CC | AA | 1 |
| S304 | Jimian11 | S3 | CC | AA | 1 |
| S305 | Jimian12 | S3 | TT | AA | 0 |
| S306 | Jimian16 | S3 | CC | AA | 1 |
| S307 | Jimian17 | S3 | TT | AA | 0 |
| S308 | Sumian4 | S3 | CC | GG | 2 |
| S309 | Ekangmian2 | S3 | TT | GG | 1 |
| S310 | Ekangmian3 | S3 | CC | GG | 2 |
| S311 | Ekangmian6 | S3 | CC | AA | 1 |
| S313 | Xuzhou142 | S2 | CC | GG | 2 |
| S315 | Sumian12 | S3 | TT | AA | 0 |
| S316 | Sukang191 | S3 | CC | AA | 1 |
| S318 | Gangmian2 | S2 | CC | AA | 1 |
| S319 | Coker 201 | S2 | CC | GG | 2 |
| S320 | Daihongdai | S2 | TT | AA | 0 |
| S323 | Kemian4 | S4 | CC | AA | 1 |
| S324 | Ganmian11 | S4 | CC | GG | 2 |
| S326 | Xianmian13 | S3 | TT | GG | 1 |
| S327 | Yapengmian | S2 | CC | AA | 1 |
| S328 | Jijiaodezimian | S1 | CC | GG | 2 |
| S329 | 611bo | S1 | TT | GG | 1 |
| S330 | Annong121 | S2 | CC | AA | 1 |
| S331 | Deltapine16 | S2 | TT | GG | 1 |
| S332 | Delfos 531 | S1 | CC | NA | 1 |
| S333 | Dunmian1 | S3 | CC | AA | 1 |
| S336 | Ganmina3 | S2 | TT | AA | 0 |
| S338 | Guangyedaizimina | S2 | CC | AA | 1 |
| S339 | Ji668 | S4 | TT | NA | 0 |
| S340 | Jimian25 | S3 | CC | AA | 1 |
| S341 | Jinmian5 | S3 | TT | AA | 0 |
| S344 | Ningmian22 | S2 | TT | GG | 1 |
| S345 | Nongda94-7 | S3 | CC | GG | 2 |
| S346 | Nongdamian8 | S4 | TT | AA | 0 |
| S347 | Shumian1 | S3 | TT | AA | 0 |
| S348 | Sumian1 | S3 | CC | GG | 2 |
| S349 | Sumian22 | S4 | TT | AA | 0 |
| S350 | Xiangmian10 | S3 | TT | AA | 0 |
| S351 | Xuzhou219 | S2 | NA | GG | 1 |
| S352 | Yinshan8 | S4 | TT | AA | 0 |
| S353 | Yumian1 | S2 | CC | AA | 1 |
| S354 | Yumian2 | S3 | TT | AA | 0 |
| S355 | Xinluzao53 | S4 | TT | AA | 0 |
| S113 | Zhong103164 | S4 | TT | AA | 0 |
| S125 | G2005 | NA | CC | GG | 2 |
| S128 | H559 | S4 | CC | GG | 2 |
| S161 | ShannongSF01 | S4 | TT | GG | 1 |
| S162 | Shan79 | NA | CC | GG | 2 |
| S163 | Stoneville 2B | S1 | CC | GG | 2 |
| S164 | Xinmian 33B | S3 | CC | GG | 2 |
| S176 | Zhong800319 | NA | CC | GG | 2 |
| S200 | Chuan65 | S4 | CC | GG | 2 |
| S204 | Xinluzao12 | S4 | TT | GG | 1 |
| S214 | Xinluzao23 | S4 | CC | GG | 2 |
| S244 | Xinluzao49 | S4 | TT | GG | 1 |
| S260 | Xinluzhong16 | S4 | NA | GG | 1 |
| S262 | Xinluzhong19 | S4 | TT | AA | 0 |
| S274 | Xinluzhong41 | S4 | TT | GG | 1 |
| S280 | Huihe36 | NA | CC | GG | 2 |
| S282 | Guoxinmian9 | S4 | CC | GG | 2 |
| S312 | Edaimian | S2 | TT | AA | 0 |
| S314 | Sumian9 | S3 | TT | AA | 0 |
| S322 | Bomian1 | S2 | CC | GG | 2 |
| S325 | Ganmian12 | S4 | CC | GG | 2 |
| S337 | Ganmian47 | S4 | CC | GG | 2 |
| S342 | Keke1543 | S2 | CC | GG | 2 |
| S343 | Ningmian1 | S2 | CC | AA | 1 |
| S277 | Xinluzhong47 | S4 | CC | GG | 2 |
