# Supplementary material for: Genome‐wide association study reveals significant loci and candidate genes for fruit branch length in upland cotton
Source: Plant Genome. 2025 May 29;18(2):e70041. doi: 10.1002/tpg2.70041 (PMC12122414; doi:10.1002/tpg2.70041)
Supplement: Supplementary file 2 [file TPG2-18-e70041-s002.docx]

**Fig. S1** Effects of two candidate genes on the fruit branch angle (FBA) of cotton accessions. (a) Violin plot for FBA of *Ghir_A10G014390*. (b) Violin plot for FBA of *Ghir_D03G011390*

**Fig. S2** Effects of *Ghir_A10G014390* on cotton fiber quality and yield. (a) Violin plot for fiber length (FL) of *Ghir_A10G014390*. (b) Violin plot for fiber elongation (FE) of *Ghir_A10G014390*. (c) Violin plot for fiber micronaire (FM) of *Ghir_A10G014390*. (d) Violin plot for fiber strength (FS) of *Ghir_A10G014390*. (e) Violin plot for fiber uniformity (FU) of *Ghir_A10G014390*. (f) Violin plot for boll weight (BW) of *Ghir_A10G014390*.

**Fig. S3.** Effects of *Ghir_D03G011390* on cotton fiber quality and yield. (a) Violin plot for fiber length (FL) of *Ghir_D03G011390*. (b) Violin plot for fiber elongation (FE) of *Ghir_D03G011390*. (c) Violin plot for fiber micronaire (FM) of *Ghir_D03G011390*. (d) Violin plot for fiber strength (FS) of *Ghir_D03G011390*. (e) Violin plot for fiber uniformity (FU) of *Ghir_D03G011390*. (f) Violin plot for boll weight (BW) of *Ghir_D03G011390*
